# Integrated head and neck imaging of symptomatic patients with stroke using simultaneous non-contrast cardiovascular magnetic resonance angiography and intraplaque hemorrhage imaging as compared with digital subtraction angiography

**DOI:** 10.1186/s12968-022-00849-1

**Published:** 2022-03-21

**Authors:** Yuxi Jia, Xiaoming Liu, Lan Zhang, Xiangchuang Kong, Shuo Chen, Lei Zhang, Jiazheng Wang, Shenglei Shu, Jia Liu, Xiaona Fu, Dingxi Liu, Jing Wang, Heshui Shi

**Affiliations:** 1 Department of Radiology, Union Hospital, Tongji Medical College, Huazhong University of Science and Technology, Wuhan, Hubei China; 2 Hubei Province Key Laboratory of Molecular Imaging, Wuhan, 430022 China; 3 Center for Biomedical Imaging Research, Tsinghua University School of Medicine, Haidian District, Beijing, China; 4 Department of Neurology, Union Hospital, Tongji Medical College, Huazhong University of Science and Technology, Wuhan, Hubei China; 5 Clinical & Technical Solutions, Philips Healthcare, Beijing, China

**Keywords:** SNAP, Stroke, Integrated head and neck vessel wall imaging, Clinical factors, Intraplaque hemorrhage, Digital subtraction angiography

## Abstract

**Background:**

Both stenosis rate and intraplaque hemorrhage (IPH) are important predictors of stroke risk. Simultaneous non-contrast angiography and intraplaque hemorrhage (SNAP) cardiovascular magnetic resonance (CMR) imaging can detect both stenosis rate and IPH. We aimed to evaluate consistency between SNAP and digital subtraction angiography (DSA) to assess symptomatic patients with stroke and explore the performance of SNAP to identify IPH and the clinical factors associated with IPH.

**Methods:**

Eighty-one symptomatic patients with stroke, admitted to Wuhan Union Hospital who underwent CMR high-resolution vessel wall imaging (HR-VWI) and SNAP, were retrospectively identified. For patients who received interventional therapy, the imaging functions of SNAP and HR-VWI were compared with DSA. The diameters of the intracranial and carotid vessels were measured, and stenotic vessels were identified. The consistency of SNAP and HR-VWI in identifying IPH was also examined, and the correlations between IPH and clinical factors were analyzed.

**Results:**

SNAP was more consistent with DSA than HR-VWI in measuring vascular stenosis (intraclass correlation coefficient [ICC]_SNAP-DSA_ = 0.917, ICC _HR-VWI-DSA_ = 0.878). Regarding the diameter measurements of each intracranial and carotid vessel segment, SNAP was superior or similar to HR-VWI, and both were consistent with DSA in the measurement of major intracranial vascular segments. HR-VWI and SNAP exhibited acceptable agreement in identifying IPH (Kappa = 0.839, 95% confidence interval [CI]: 0.704–0.974). Patients who underwent interventional therapy had a higher plaque burden (P < 0.001). Patients with IPH had lower levels of high-density lipoprotein cholesterol (HDL) (P = 0.038) and higher levels of blood glucose (P = 0.007) and cystatin C (P = 0.040).

**Conclusions:**

CMR SNAP is consistent with DSA in measuring vessel diameters and identifying atherosclerosis stenosis in each intracranial and carotid vessel segment. SNAP is also a potential alternative to HR-VWI in identifying stenosis and IPH.

**Supplementary Information:**

The online version contains supplementary material available at 10.1186/s12968-022-00849-1.

## Introduction

Intracranial and carotid atherosclerotic disease are the major causes of ischemic stroke worldwide, especially in Asia [1]. The risk assessment of ischemic stroke is based on the percentage of stenotic intracranial and carotid arteries, according to medical imaging findings [2, 3]. Digital subtraction angiography (DSA) is the gold standard for evaluating arterial stenoses. However, it has many disadvantages, including being invasive, costly, and complicated to perform. Cardiovascular magnetic resonance (CMR) high-resolution vessel wall imaging (HR-VWI), which provides a non-invasive and clear visualization of vessel lumens and walls, has been used increasingly in the study of atherosclerotic plaques. HR-VWI has been reported to provide significant data for the diagnosis, clinical treatment, and prognosis of patients with stroke [4–6]. As a new CMR-based technique, simultaneous non-contrast angiography and intraplaque hemorrhage (SNAP) imaging can show the presence of both lumen and intraplaque hemorrhage (IPH) simultaneously with a wide field-of-view (FOV) that covers large vessels in both the head and the neck [7]. Recent studies have assessed the ability of HR-VWI and SNAP to characterize atherosclerotic plaque compositions, stenoses, and their association with ischemic stroke. The imaging functions of these modalities were also compared with those of conventional vascular imaging techniques [8–12]. Previous research suggested that SNAP was efficient at identifying IPH and had satisfactory agreement with histopathology [8]. Several current guidelines recommend HR-VWI as the most appropriate diagnostic method to assess intracranial and extracranial atherosclerotic plaques, especially vulnerable plaques [13], and SNAP is suggested as a complement to identify vulnerable plaque components, mainly for IPH [14]. Compared with SNAP, HR-VWI examinations including multi-sequence imaging, are more time-consuming. If the diagnostic performance of SNAP and HR-VWI is similar to DSA in measuring the stenosis severity, SNAP could replace HR-VWI in some clinical circumstances and might improve diagnostic efficacy.

A growing body of evidence suggests that the composition and burden of plaques are important for stroke assessment and prognosis [13, 15]. IPH, as a plaque component, is particularly important for the prediction of plaque progression [16]. Previous literature demonstrate that the early detection of IPH enables clinicians to determine whether patients can benefit from carotid endarterectomy or stenting, which could eventually improve the prognosis of patients with stroke [17]. Exploring IPH-associated clinical factors via routine clinical biochemical examinations may remind clinicians to pay attention to the existence of IPH before an imaging examination, leading to diagnostic efficacy improvements.

There are few studies comparing the performance of SNAP and DSA in assessing arterial stenosis in the head and neck vessels, as well as the association between IPH and clinical factors. The present study aimed to compare the performance of SNAP with DSA in measuring luminal stenosis of the head and neck arteries. It also explored the value of SNAP in identifying IPH and the clinical factors associated with IPH to provide an effective imaging basis for improving diagnostic and therapeutic efficacy.

## Methods

### Study population

**Fig. 1:**
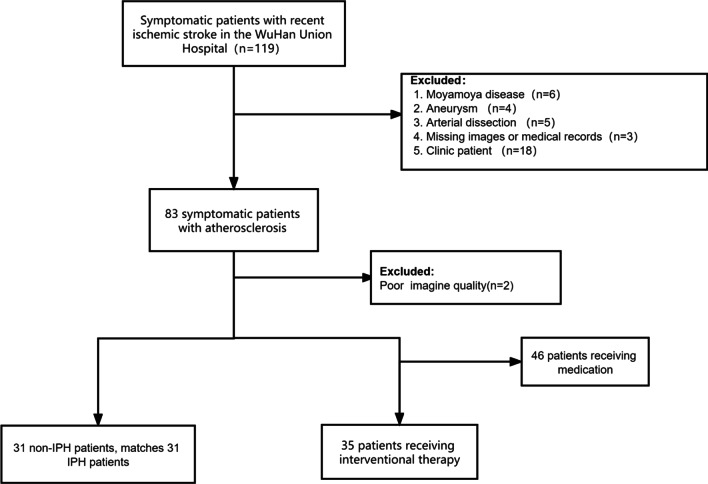
Flow chart of the study design

Data from symptomatic patients with stroke, admitted to the Union Hospital Affiliated with Tongji Medical College of Huazhong University of Science and Technology (Wuhan, China) from January 2019 to January 2020, were retrospectively analyzed (Fig. 1). The study was approved by the Medical Ethics Committee of Union Hospital affiliated with Tongji Medical School of Huazhong University of Science and Technology (Approval No. 2021-IEC133). Written informed consent was waived.

Inclusion criteria included: (1) symptomatic inpatients (18 to 80 years); (2) recent stroke (within the first two weeks after admission); (3) underwent both SNAP and HR-VWI, with or without interventional therapy (e.g., mechanical thrombectomy). Exclusion criteria were: (1) non-atherosclerotic causes of stroke, such as Moyamoya disease, vasculitis, and arterial dissection; (2) hemorrhagic stroke; (3) cerebral neoplasm; (4) cardiogenic stroke (e.g., atrial fibrillation, heart failure, or myocardial infarction); (5) poor-quality CMR images; (6) contraindications for gadolinium-based contrast agent administration.

### Data collection

Clinical data were extracted from the patient’s electronic medical records, including age, sex, height, weight, body mass index (BMI), systolic blood pressure (SBP), diastolic blood pressure (DBP), and pulse pressure (PP). Traditional vascular risk factors, including hypertension, smoking, alcohol, diabetes, hyperlipidemia, and levels of total protein, serum amyloid A (SAA), total cholesterol, high-density lipoprotein cholesterol (HDL), low-density lipoprotein cholesterol (LDL), and triglycerides were also obtained. All biochemical markers mentioned in our study were measured using routine biochemical tests within two weeks of admission.

### Imaging examination

Patients underwent vessel wall imaging on a 3 T CMR system (Ingenia CX, Philips Healthcare, Best, the Netherlands) with a combination of a 32-channel head coil (Philips Healthcare) and an 8-channel neck coil (TSImaging Healthcare, Beijing, China). The imaging protocols are summarized as follows: 3-dimensional (3D) time-of-flight (3D-TOF), 3D SNAP in both transverse and coronal orientations, multi-sequence HR-VWI (e.g., coronal 3D T1-weighted variable flip angle fast spin-echo sequences [VISTA; Philip Healthcare] before and after contrast administration, and coronal T2-weighted (T2w) VISTA), T1-weighted inversion recovery (T1w-IR), T2-FLAIR-weighted (T2w-FLAIR), and diffusion-weighted-imaging (DWI). 3D-TOF, T1w, T2w-FLAIR, and DWI were used to identify symptomatic patients with stroke. 3D-TOF imaging was used to detect arterial stenosis. Moreover, the T1w imaging, T2w-FLAIR, and DWI were used to find infarcted intracranial lesions. We also used 3D SNAP, 3D T1-VISTA, and 3D T2-VISTA to assess both intracranial and extracranial arterial stenosis and plaque. The T1/T2-VISTA sequences were used to identify the atherosclerotic plaques and their compositions, and the contrast-enhanced T1-VISTA imaging to further determined the nature of the plaque and to measure the degree of stenosis.

The main imaging parameters were as follows: For 3D TOF CMR angiogram (CMRA): fast field echo (FFE) sequence, repetition time (TR)/echo time (TE) = 23/3.5 ms; flip angle (FA) = 18°; field of view (FOV) = 160 × 160 mm^2^; slices = 150; slice thickness = 0.55 mm; acquisition matrix size = 500 × 332; reconstruction voxel size = 0.4 × 0.4 × 0.55 mm^3^; parallel acquisition factor = 3; and scan time = 4 min and 15 s. For T1w imaging: turbo spin echo (TSE) sequence, TR/TE = 2000/16 ms; acquisition matrix size = 288 × 204; slice thickness = 5 mm. For T2w-FLAIR: TSE sequence, TR/TE = 9000/125 ms, acquisition matrix size = 384 × 192; slice thickness = 5 mm. For DWI: spin echo (SE) sequence, TR/TE = 2732/77 ms; acquisition matrix size = 152 × 106; FA = 90°; slices = 23; slice thickness = 5 mm. DWI was obtained with b values of 0 and 1000 s/mm2.

For T1w VISTA: TSE sequence, TR/TE = 600/26 ms; FA = 90°; acquisition matrix = 416 × 270; slice thickness = 0.6 mm; reconstruction voxel size = 0.6 × 0.6 × 0.6 mm^3^; compressed sensing factor = 3; scan time = 4 min and 52 s. For T2wVISTA: TSE sequence, TR/TE = 1800/214 ms; FA = 90°; acquisition matrix = 416 × 270; slice thickness = 0.6 mm; reconstruction voxel size = 0.6 × 0.6 × 0.6 mm^3^; compressed sensing factor = 3; and with SPAIR fat suppression, scan time = 4 min and 26 s. For SNAP: FFE sequence, TR/TE = 11/5.9 ms; FA = 11°; inversion time = 500 ms; FOV = 160 × 160 mm^2^; slices = 150, slice thickness = 0.8 mm; acquisition matrix size = 200 × 200; reconstruction voxel size = 0.4 × 0.4 × 0.4 mm^3^; parallel acquisition factor = 2; scan time = 3 min and 2 s.

### Data analysis

Two radiologists (Y.J. and X.L., with 2 and 5 years of experience in neuro-vascular imaging, respectively) analyzed the imaging examinations results independently using Vessel Explorer 2.0 software (TS Imaging Healthcare, Beijing, China). Both were blinded to patients' clinical characteristics. HR-VWI and SNAP assessments were performed independently, and the interval between the two radiologists’ assessments was about one month. The results of HR-VWI/SNAP were also evaluated independently against the DSA results, and the evaluation interval between the HR-VWI/SNAP and DSA was also about one month. In addition, both radiologists also investigated the location and number of IPH on the SNAP images. The lumen, wall, and IPH components of SNAP and HR-VWI were manually delineated [18, 19].

Disagreements were resolved by discussion between the two radiologists. If an agreement could not be reached, a senior radiologist (J.W. with 12 years of neuro-radiology experience) would repeat the measurements and come to a consensus.

Multi-contrast vessel wall imaging of arteries of the head and neck arteries (T1w-VISTA, T2w-VISTA, and CE-T1w-VISTA) was reconstructed using the software to measure luminal stenosis via the North American Symptomatic Carotid Endarterectomy Trial (NASCET) algorithm or the Warfarin-Aspirin Symptomatic Intracranial Disease Study (WASID) algorithm [20, 21]. The common carotid artery (CCA), internal carotid arteries (C1-7), M1 segment of middle cerebral arteries, V4 segment of vertebral arteries, and basilar artery on these sequences were analyzed. The degree of stenosis was defined as: (1) mild, < 50%; (2) moderate, 50–70%; and (3) severe, 70% ~ 99% [4]. The stenosis was defined as a decrease in the diameter of the lumen as a result of an eccentric or circumferential thickening of the vessel wall compared to the image slices obtained from beneath or above the focal wall, as identified on the SNAP or HR-VWI. Arterial stenotic rates were measured using DSA with the following equation: 1- the diameter of the narrowest part / (the mean of the normal diameter on both sides). Stenosis measurements on SNAP/HR-VWI were as follows: (1—minimum luminal diameter/normal reference diameter) × 100%. The IPH was defined as the presence of increased T1 signal intensity (> 150%) on the T1w-VISTA or SNAP sequence as compared with the adjacent grey matter or the sternocleidomastoid muscle [22]. Plaque burden = (1-lm/wall area) × 100% [23].

### Statistical analysis

SPSS (version 22.0, Statistical Package for the Social Sciences, International Business Machines, Inc., Armonk, New York, USA) was used for statistical analyses. Continuous variables are presented as the mean ± standard deviation (SD), and categorical variables are presented as the frequency and percentage. For clinical factor analyses, an unequal number of patients with or without IPH was matched according to the age distribution and sex ratio of the two groups. The Chi-square and McNemar’s tests were used to assess associations between the presence of IPH and vascular risk factors. Nonparametric test was used to investigate the relationship between the presence of IPH and clinical data when the data were abnormally distributed. The intraclass correlation coefficient (ICC) and Bland–Altman plot with 95% confidence intervals (CIs) were calculated to evaluate intraclass and intergroup reproducibility in diagnosing the degrees of stenosis. The Kappa value was used to evaluate the consistency between SNAP and HR-VWI in identifying IPH. Statistical significance was set at P < 0.05.

## Results

### Patient characteristics at baseline

Eighty-one symptomatic patients with stroke were identified (Table 1) (54.6 ± 9.5 years; 67 (82.7%) male). Among them, 35 patients underwent DSA-guided interventional therapy, who demonstrated a relatively higher plaque burden (P < 0.001) (vs. medication therapy). However, patients treated with different methods had no significant difference in other aspects, including age, sex, and complications (all P > 0.05) (Table 1). Patients with and without IPH were matched by age and sex. For the clinical factors, patients with IPH were not statistically different in age, height, systolic and DBP, and pulse pressure (Table 2). Patients with or without IPH also did not have significant differences in serum total protein, SAA, total cholesterol, LDL, triglycerides, and homocysteine concentrations (P > 0.05). The blood glucose (P = 0.007) and cystatin C (P = 0.04) concentrations were higher in IPH patients. In contrast, patients with IPH had lower HDL (P = 0.038). No significant differences in plaque burden (89.7 ± 9.6, P > 0.05), hypertension (n = 24 (77.4% vs. 54.8%), P = 0.06), or other comorbidities were found between patients with or without IPH.

**Table 1:** Comparison of patient data at baseline

	Mean ± SD or N (%)			P-value
	All patients (N = 81)	Patients receiving medication (n = 46)	Patients receiving interventional therapy (n = 35)	
Age, years	54.6 ± 9.5	53.5 ± 12.3	53.7 ± 10.2	0.731
Sex, Male	67 (82.7%)	38 (82.6%)	29 (82.9%)	0.977
BMI, kg/m^2^	24.5 ± 3.8	24.2 ± 2.4	23.7 ± 2.9	0.913
PP, mmHg	55.8 ± 16.1	55.6 ± 19.2	58.4 ± 14.9	0.312
Plaque Burden	94.7 ± 3.0	86.9 ± 9.7	94.9 ± 2.6	< 0.001
Smoking	36 (46.8%)	21 (47.7%)	15 (44.1%)	0.751
Drinking	30 (38.5%)	15 (34.1%)	15 (38.5%)	0.367
Hypertension	55 (68.8%)	31 (68.9%)	24 (68.6%)	0.976
Cardiovascular disease	27 (33.8%)	12 (26.7%)	15 (42.9%)	0.129
Hyperlipidemia	23 (28.7%)	12 (26.7%)	11 (31.4%)	0.641
Diabetes	29 (36.3%)	15 (33.3%)	14 (40%)	0.538
Hyperuricemia	6 (7.5%)	5 (11.1%)	1 (2.9%)	0.223

*BMI* body mass index, *PP* pulse pressure

**Table 2:** Comparison of patient data with or without intraplaque hemorrhage (IPH)

	Mean ± SD/N (%)			P-value
	All patients (n = 62)	Non-IPH patients (n = 31)	IPH patients (n = 31)	
Age, years	54.9 ± 10.8	53.5 ± 11.9	56.2 ± 9.7	0.336
Sex, Male	67 (82.7%)	16 (88.9%)	51 (81%)	1.000
Height, cm	168.0	167.6 ± 5.9	168.3 ± 6.4	0.787
Weight, kg	69.3 ± 9.5	68.6 ± 8.0	68.7 ± 9.8	0.783
BMI, kg/m^2^	24.6 ± 2.7	24.5 ± 2.8	24.2 ± 2.8	0.986
SBP, mmHg	141.8 ± 17.2	137.4 ± 15.3	146.7 ± 16.1	0.285
DBP, mmHg	86.7 ± 10.3	84.6 ± 11.6	88.6 ± 9.9	0.254
PP, mmHg	55.1 ± 16.3	52.8 ± 14.5	58.1 ± 15.6	0.683
TP, g/L (64–83)	64.1 ± 4.9	63.1 ± 4.0	64.2 ± 4.7	0.127
Total cholesterol, mmol/L(< 5.2)	3.5 ± 0.7	3.6 ± 0.6	3.4 ± 0.8	0.509
HDL, mmol/L (1.16–1-42)	1.1 ± 0.3	1.1 ± 0.3	1.0 ± 0.3	0.038*
LDL, mmol/L (2.7–3.1)	1.9 ± 0.7	1.9 ± 0.7	1.8 ± 0.7	0.593
Triglycerides, mmol/L (< 1.7)	1.6 ± 1.1	1.6 ± 1.2	1.6 ± 1.0	0.079
Glucose, mmol/L (3.9–6.1)	5.8 ± 1.7	5.2 ± 1.0	6.1 ± 1.6	0.007*
Cystatin C, mg/L (0.63–1.25)	0.93 ± 0.33	0.83 ± 0.18	1.05 ± 0.46	0.040*
Homocysteine (< 20 μmol/L)	11.4 ± 10.0	13.2 ± 15.4	9.9 ± 3.5	0.846
SAA (< 10.0 mg/L)	15.0 ± 36.7	5.4 ± 4.2	13.6 ± 17.0	0.117
Degree of stenosis				
Mild, < 50%	13 (20.97%)	9 (29%)	4 (12.9%)	0.196
Moderate, 50–70%	21 (33.9%)	11 (35.5%)	10 (32.3%)	
Severe, 70–99%	28 (45.2%)	11 (35.5%)	17 (54.8%)	
Plaque Burden	88.8 ± 9.5	86.8 ± 8.0	89.7 ± 10.0	0.09
Smoking	24 (38.7%)	11 (50.0%)	13 (41.9%)	0.561
Drinking	22 (35.5%)	7 (31.8%)	15 (48.4%)	0.228
Hypertension	41 (66.1%)	17 (54.8%)	24 (77.4%)	0.06
Cardiovascular disease	20 (32.3%)	8 (25.8%)	12 (38.7%)	0.227
Hyperlipidemia	16 (25.8%)	7 (22.6%)	9 (29.0%)	0.562
Diabetes	17 (27.4%)	6 (19.4%)	11 (35.5%)	0.155
Hyperuricemia	8 (12.9%)	6 (19.4%)	2 (6.5%)	0.13

*BMI* body mass index, *SBP* systolic blood pressure, *DBP* diastolic blood pressure, *PP* pulse pressure, *TP* total protein, *HDL* high-density lipoprotein cholesterol, *LDL* low-density lipoprotein cholesterol, *IPH* intraplaque, hemorrhage, *SAA* serum amyloid A, * P < 0.05

### Performance of SNAP in identifying IPH

The location and number of IPH were counted in patients with IPH (Table 3). A total of 52 IPH lesions were identified in the intracranial arteries of 31 patients, and 26 IPH lesions were found in the carotid arteries. IPH was detected in both carotid and intracranial locations. Six of the 31 patients had IPH in both the neck and intracranial regions. Seventeen patients had multiple IPH lesions in intracranial or carotid arteries. A greater number of IPH lesions were detected in the major intracranial vessels than in the carotid artery. SNAP and HR-VWI had good consistency in identifying IPH (Table 4, Kappa = 0.839). In identifying IPH, SNAP had a similar consistency in identifying IPH in intracranial and carotid arteries (Additional file 1: Table S1). Four patients with IPH had mild stenosis (12.9%), 10 had moderate stenosis (32.3%) and 17 had severe stenosis (54.8%). There was no significant difference in the degree of stenosis between the patients with and without IPH. However, the proportion of patients with IPH increased with the degree of stenosis among the patients with different degrees of stenosis (4/13 [30.77%] mild vs. 10/21 [47.62%] moderate vs.17/28 [60.71%] severe).

**Table 3:** The location and number of intraplaque hemorrhage (IPH) lesions found on the simultaneous non-contrast angiography and intraplaque hemorrhage (SNAP) images

IPH		
Location	Number	Percent (%)
Intracranial vessel	52	66.7
MCA	40	51.2
BA	5	6.4
V4	7	8.9
Carotid	26	33.3
CCA	2	2.6
C1	6	7.7
C2	2	2.6
C3	1	1.3
C4	3	3.9
C5	2	2.6
C6	4	5.1
C7	6	7.7

*IPH* intraplaque hemorrhage, *MCA* middle cerebral artery, *BA* basilar artery, *CCA* common carotid artery

**Table 4:** Agreement between simultaneous non-contrast angiography and intraplaque hemorrhage (SNAP) imaging and high-resolution vessel wall imaging (HR-VWI) in identification of intraplaque hemorrhage (IPH)

		HR-VWI		Cohen's kappa	95% CI of kappa	P
		Absence	Presence			
SNAP	Absence	30	1	0.839	0.704–0.974	< 0.001
	Presence	4	27			

*SNAP* simultaneous non-contrast angiography and intraplaque hemorrhage, *HR-VWI* high-resolution vessel wall imaging, *IPH* intraplaque hemorrhage

### Comparison of the functions of SNAP and DSA in the measurement of luminal stenosis

**Fig. 2:**
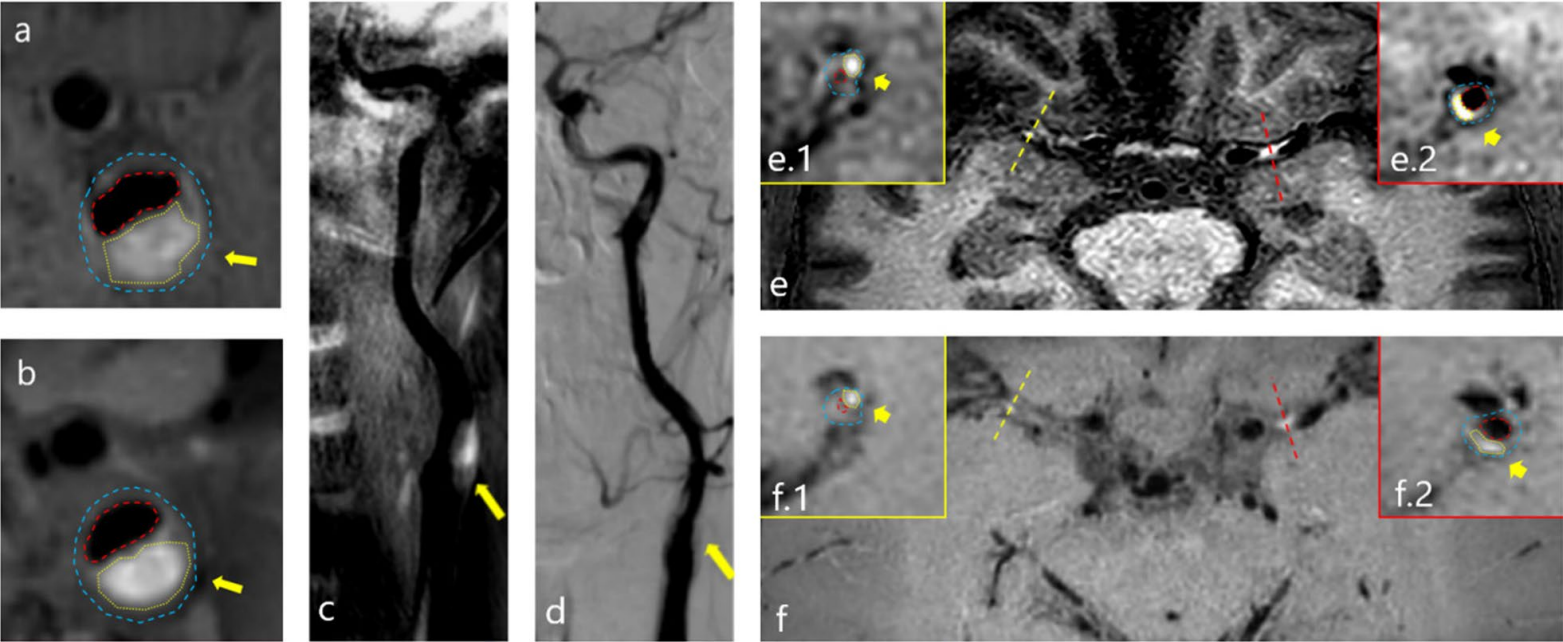
Images from typical simultaneous non-contrast angiography and intraplaque hemorrhage (SNAP) imaging and high-resolution vessel wall imaging (HR-VWI) in symptomatic patients with stroke. Images **a**, **b** represent the SNAP and T1-VISTA images of a 43-years-old man with acute stroke. Eccentric plaques (narrow yellow arrow) were observed at the beginning of the C1 segment of the left internal carotid artery (ICA) of this patient. Images **c**, **d** show the corresponding coronal SNAP and digital subtraction angiography (DSA) images. Images **e**, **f** images are the SNAP and T1-VISTA images from a 50-years-old man with subacute stroke and with IPH plaques in the right side (images **e.1** and **f.1**) and the left side (images **e.2** and **f.2**) of the middle carotid artery (MCA; wide yellow arrow). The blue dots and lines indicate vessel walls. The red dots and lines represent vessel lumens. The yellow dots and lines indicate the presence of IPH

**Fig. 3:**
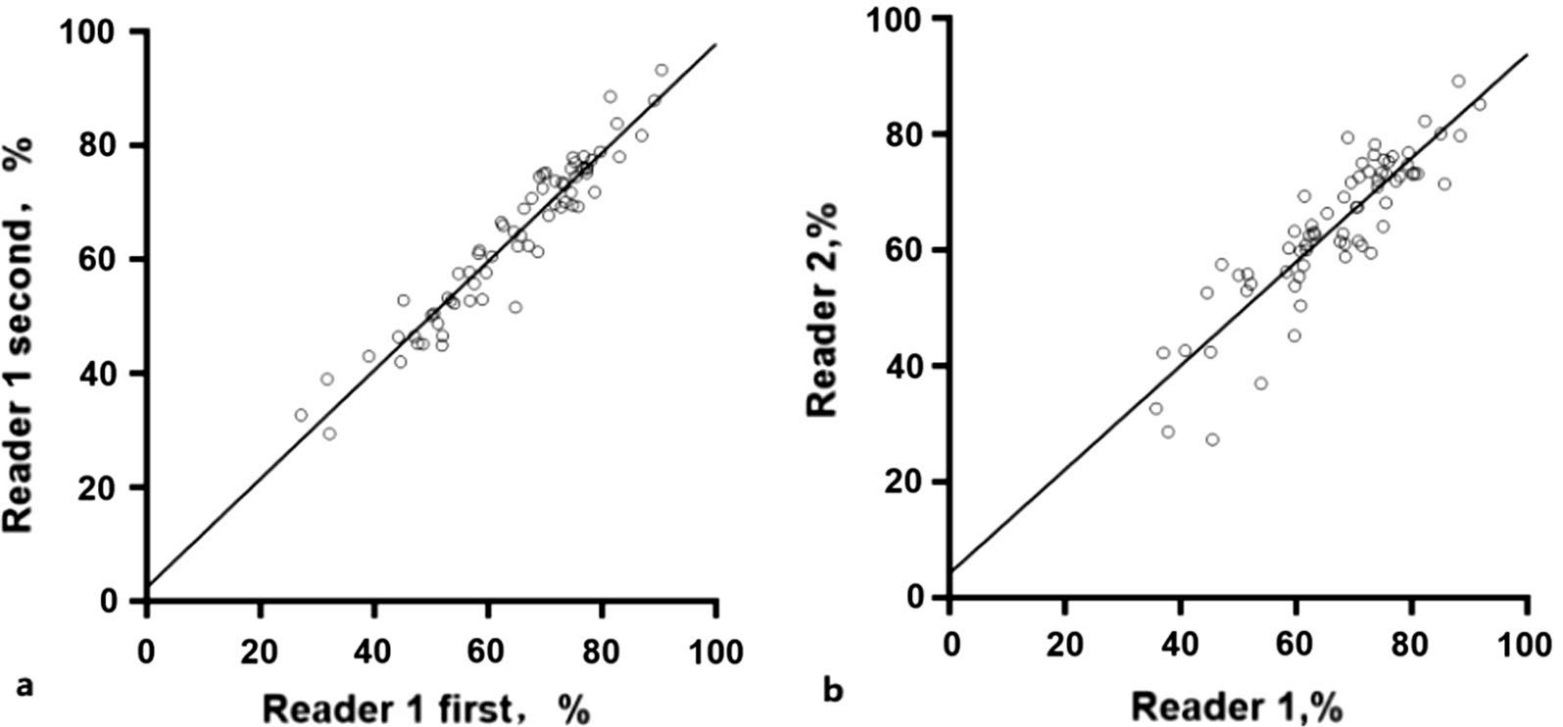
The intraclass correlation coefficient (ICC) values examined the same reader at different times and between the two readers. **a** Good consistency was found for the assessments by the same reader at different times(ICC_intraclass_ = 0.960). **b** Better consistency was found between the two reader assessments in measuring the degree of stenosis (ICC_interclass_ = 0.889)

**Fig. 4:**
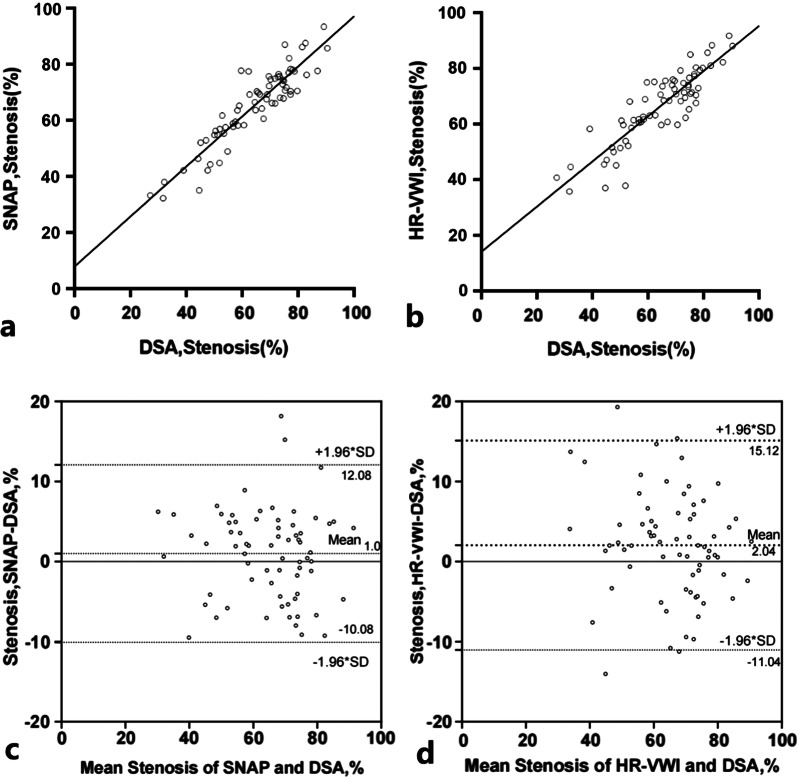
Simultaneous non-contrast angiography and intraplaque hemorrhage (SNAP) imaging with digital subtraction angiography (DSA) typically have a better consistency than HR-VWI with DSA. **a** ICC_SNAP-DSA_ = 0.917, **b** ICC_HR-VWI-DSA_ = 0.878, **c**, **d** Bland–Altman plots comparing SNAP and DSA vs. HR-VWI and DSA

**Fig. 5:**
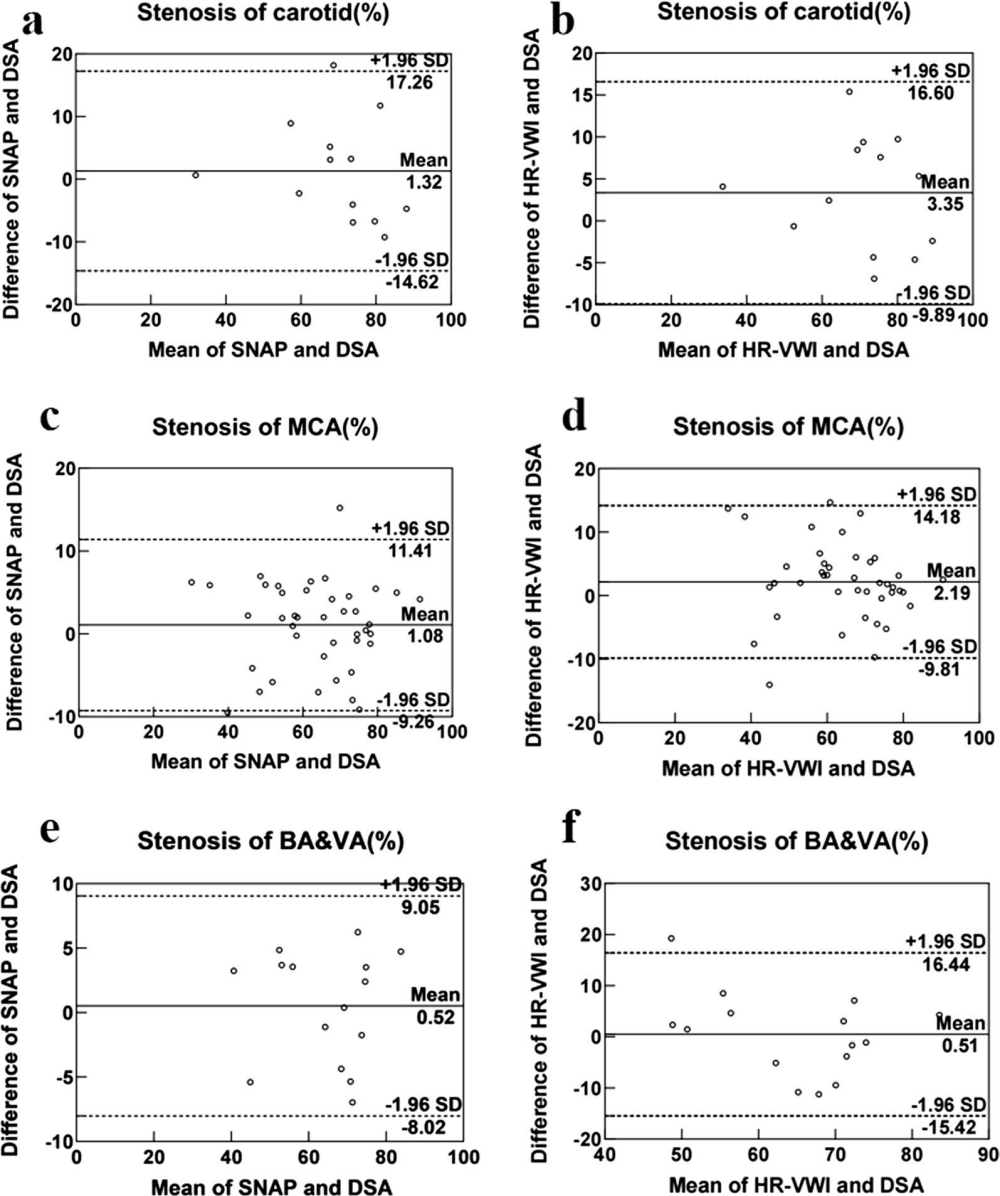
Bland–Altman plots comparing the bias of simultaneous non-contrast angiography and intraplaque hemorrhage (SNAP) and high-resolution vessel wall imaging (HR-VWI) images in measuring different stenotic segments of intracranial and carotid arteries using DSA images as the reference images. (*ICA* internal carotid artery, *MCA* middle cerebral artery, *BA* basilar artery, *VA* vertebral artery)

Thirty five patients underwent both HR-VWI and DSA-guided interventional therapy, and 69 luminal stenosis sites and plaques were evaluated using both SNAP and DSA (Fig. 1–2). Consistency of the same reader at different times (ICC_intraclass_ = 0.960) and between the two readers (ICC_interclass_ = 0.889) were satisfactory (Fig. 3 a & b). Moreover, SNAP (ICC_SNAP-DSA_ = 0.917) and HR-VWI (ICC_HR-VWI-DSA_ = 0.878) both showed good agreement with DSA the measurement of luminal stenosis (Fig. 4). Of the 69 stenotic segments detected by integrated head and neck imaging, 14 were in the carotid artery, and 55 were in the intracranial artery. We found that SNAP and HR-VWI had acceptable consistency with DSA according to each segment of artery stenosis (Fig. 5). Then, we evaluated SNAP, HR-VWI and DSA in each segment of the intracranial and carotid arteries, including normal and abnormal segments, and found that SNAP and HR-VWI had satisfactory consistency with DSA in each segment (Additional file 1: Fig. S1). SNAP was superior or similar to HR-VWI and was consistent with DSA in the major intracranial vascular segments.

## Discussion

Although the primary indicator of ischemic stroke risk is the rate of stenosis, plaque components are becoming increasingly recognized as important in assessing stroke risk, as shown in the current study. IPH is an important risk factor for stroke compared with other plaque components [24]. IPH is common in asymptomatic and symptomatic patients with stroke, while symptomatic patients are at a greater risk for IPH than asymptomatic patients [25]. In the present study, we studied the symptomatic patients with stroke and investigated whether SNAP and HR-VWI were well-aligned with DSA for measuring the stenosis and diameter of both intracranial and carotid arteries. We also studied if SNAP was similar to HR-VWI in being able to detect IPH. The results showed that SNAP was more consistent with DSA at measuring luminal stenosis than HR-VWI. When measuring general segments, SNAP was also better than or similar to HR-VWI in the measurement of intracranial vascular segments. Additionally, SNAP had good inter-group consistency for the intracranial and carotid arteries and a good consistency with HR-VWI in identifying IPH. Based on these results, we analyzed IPH-related clinical factors. The main clinical factors associated with IPH were HDL, glucose, and cystatin C.

SNAP performance in detecting luminal stenosis has been studied previously [8–12]. Wang et al. reported that SNAP had a good agreement with time-of-flight (TOF)-CMR angiography (TOF-CMRA) in measuring carotid artery lumens [8]. Shu et al. showed that, compared with CE-CMRA, SNAP was equally capable of measuring the carotid artery stenosis [9]. Several scholars have also suggested that SNAP had good consistency with TOF-MRA, magnetization prepared-rapid gradient echo (MPRAGE), and histology in characterizing plaque components, especially for IPH detection [10, 11]. SNAP was also shown to be consistent with HR-VWI in characterizing large intracranial vessels [12], while HR-VWI was found to have acceptable agreement with DSA in measuring the diameter of each carotid artery segment [7]. However, few studies have concentrated on the integrated imaging of intracranial and carotid vessel walls [26, 27], and no study has comprehensively assessed the imaging performance of SNAP and DSA on head and neck vascular walls. Our results showed that SNAP and HR-VWI were consistent with DSA in each intracranial and carotid vessel segment, and the results were reproducible and repeatable. SNAP is a non-contrast-enhanced integrated head and neck imaging technique that can be used for a wider range of individuals than conventional high-resolution imaging and DSA, the gold standard imaging technique, especially for contrast-sensitive patients and critically ill patients with poor liver and kidney functions.

To the best of our knowledge, CMR can evaluate IPH easier and faster than other plaque components. CMR is also more amenable to in-clinic evaluation. SNAP and HR-VWI were all found to be in good agreement with pathologic findings in the identifying IPH in the previous literatures [11, 28]. In the current study, we found that SNAP and HR-VWI had good consistency in the identifying of IPH, and no significant difference in the number of IPH and non-IPH patients among those with different degrees of stenosis, which could be related to the relatively small sample size or a potentially complicated relationship between stenosis and various plaque components [29, 30]. Additionally, mild stenosis might be partly caused by plaque remodeling [31]. Therefore, the number of IPH lesions was observed to increase as the degree of stenosis increased; however, no significant correlation was found between stenosis and the presence of IPH lesions, indicating the study of plaque remodeling is needed. But, overall, we found that as the degree of stenosis increased, the presence of IPH also increased. Our study included the assessment of both carotid and intracranial arteries, and we came to a similar conclusion as those of a previous study [32]. That study demonstrated an increase in the degree of carotid artery stenosis independently associated with the presence of IPH. IPH was detected in both carotid and intracranial vessels. Moreover, IPH was detected in the major intracranial vessels more often than in the carotid artery. Therefore, the integration of head and neck imaging can help detect and localize more IPH lesions, which is helpful for diagnosing etiologies, giving treatment guidance, and providing prognostic evaluations for symptomatic patients with stroke. SNAP is a one-stop solution for measuring narrowness and identifying IPH and has a shorter scan time than HR-VWI. However, it is worth noting that the bright signal in the T1w intracranial artery images in our study was defined as IPH with no histological evidence provided, which is a limitation of the study. On the other hand, this image interpretation is similar to previous radiological studies on intracranial IPH [33, 34] and has been supported by some specimen studies [28].

A previous study found that IPH in carotid plaques using MPRAGE imaging was associated with age, sex, degree of stenosis, and hyperlipidemia [35], which was, not observed in our study. This may be related to the cohort characteristics where patients with primarily moderate to severe arterial stenosis were included; some also had partial occlusions. In addition, rather than considering carotid plaques, we included all intracranial and carotid arterial plaques that could have influenced the analytic results, owing to the high incidence of intracranial plaques in Chinese patients and the high incidence of extracranial plaques in European and American patients [36]. A comprehensive assessment of the presence of plaques enables us to identify the etiology of stroke better.

The specific relationship between cystatin C and IPH has not been previously reported. Cystatin C is an inhibitor of elastase that can affect the extracellular matrix remodeling of atherogenesis. Although cystatin C levels could also indicate renal dysfunction, a number of scholars have reported the association of higher cystatin C levels,with an increased risk of cardiovascular-related death [37], a higher degree of intracranial and extracranial artery stenosis, and the presence of vulnerable plaques in patients with acute ischemic stroke [38, 39]. Increased serum cystatin C levels have also been shown to be associated with subclinical atherosclerosis and recurrent stroke [40, 41]. Therefore, increased serum cystatin C levels could be related to the progression of arteriosclerosis plaque. In the present study, we showed that patients with IPH also tended to have significantly higher serum cystatin C levels than those without IPH. But our study had a small sample size, we still need further research in the future.

Diabetes is known to play an important role in stroke. The Rotterdam Study showed that IPH is associated with increased serum insulin levels but not with increased glucose levels [42]. Previous studies have also found that diabetes was highly associated with lipid-rich necrotic cores (LRNC) of plaques and that the volume ratio of LRNC > 22.0% in carotid plaques could be an independent risk factor for acute cerebral infarction [43]. In our study, although the glucose levels were significantly different between the IPH and non-IPH groups, diabetes was not found to be significantly associated with IPH. We speculate that the varying glucose levels were a result of the varying serum insulin levels.

Hypertension is an important risk factor for clinical cardiovascular events. A study [44] on carotid arteries found that increases in both systolic and diasotlic blood pressure were associated with a range of plaque components, including IPH. Hypertension also affected vessel wall volume and fibrous cap. Hypertension may contribute to fibrous cap rupture and IPH, but we did not observe a similar result, perhaps due to the small sample size. Low diastolic blood pressure has been found to be associated with IPH in patients with asymptomatic atherosclerosis [45]; however, the underlying mechanism needs further study in the future.

## Limitations

This study has several limitations. First, our study had a small sample size and was a single-center study. Second, there might have been some bias in our population selection, which primarily selected for symptomatic patients with moderate to severe stroke. Third, we did not have histopathologic results of plaque as the gold standard to identify IPH, especially in the intracranial arteries. Finally, a small percentage of the clinical data were missing due to the retrospective nature of this study; we attempted to decrease the influence of missing data on the conclusions as much as possible. Therefore, the conclusions should be considered carefully when generalizing the findings.

## Conclusion

Among patients with recent symptomatic stroke, CMR SNAP is consistent with DSA in measuring the diameter of each intracranial and carotid arterial segment and stenotic atherosclerotic lesions. SNAP was also demonstrated a potential alternative to HR-VWI in identifying stenosis and IPH.

## Supplementary Information

**Additional file 1. Table S1. **The inter-group consistency of the simultaneous non-contrast angiography and intraplaque hemorrhage (SNAP) imaging in identifying intraplaque hemorrhage (IPH) at different sites of intracranial and carotid arteries.** Fig. S1** The agreement between simultaneous non-contrast angiography and intraplaque hemorrhage (SNAP) and high-resolution vessel wall imaging (HR-VWI) with digital subtraction angiography (DSA) in measuring the diameter of different intracranial and carotid arterial segments.

## Data Availability

The datasets used or analyzed during the current study are available from the corresponding authors on reasonable request.

## Abbreviations

3D: Three dimensional
BMI: Body mass index
CCA: Common carotid artery
CMR: Cardiovascular magnetic resonance
CMRA: Cardiovascular magnetic resonance angiography
DBP: Diastolic blood pressure
DSA: Digital subtraction angiography
DWI: Diffusion weighted imaing
FFE: Fast field echo
FOV: Field-of-view
HDL: High-density lipoprotein
HR-VWI: High resolution vessel wall imaging
ICA: Internal carotid artery
IPH: Intraplaque hemorrhage
LDL: Low density lipoprotein
LRNC: Lipid rich necrotic core
MPRAGE: Magnetization prepared-rapid gradient echo
NASCET: North American Symptomatic Carotid Endarterectomy Trial
PP: Pulse pressure
SAA: Serum amyloid A
SBP: Systolic blood pressure
SNAP: Simultaneous non-contrast angioraphy and intraplaque hamorrhage
TI: Inversion time
TOF: Time-of-flight
TP: Total protein
TSE: Turbo spin echo
