# Supplementary material for: Integrated head and neck imaging of symptomatic patients with stroke using simultaneous non-contrast cardiovascular magnetic resonance angiography and intraplaque hemorrhage imaging as compared with digital subtraction angiography
Source: J Cardiovasc Magn Reson. 2022 Mar 21;24:19. doi: 10.1186/s12968-022-00849-1 (PMC8935695; doi:10.1186/s12968-022-00849-1)

**Additional file 1: Table S1.** The inter-group consistency of the simultaneous non-contrast angiography and intraplaque hemorrhage (SNAP) imaging in identifying intraplaque hemorrhage (IPH) at different sites of intracranial and carotid arteries.

| Intracranial artery of patients with IPH | | SNAP2 | | Cohen's kappa | 95%CI of kappa | P |
| --- | --- | --- | --- | --- | --- | --- |
|  |  | Absence | Presence |  |  |  |
| SNAP1 | Absence | 10 | 3 | 0.794 | 0.602-0.986 | <0.001 |
|  | Presence | 1 | 49 |  |  |  |

| Carotid of patients with IPH | | SNAP2 | | Cohen's kappa | 95%CI of kappa | P |
| --- | --- | --- | --- | --- | --- | --- |
|  |  | Absence | Presence |  |  |  |
| SNAP1 | Absence | 11 | 1 | 0.821 | 0.645-0.997 | <0.001 |
|  | Presence | 2 | 24 |  |  |  |

SNAP1, the first radiologist; SNAP2, the second radiologist.

**Additional file 1: Fig. S1** The agreement between simultaneous non-contrast angiography and intraplaque hemorrhage (SNAP) and high-resolution vessel wall imaging (HR-VWI) in measuring the diameter of different intracranial and carotid arterial segments.(A-B)

(A)


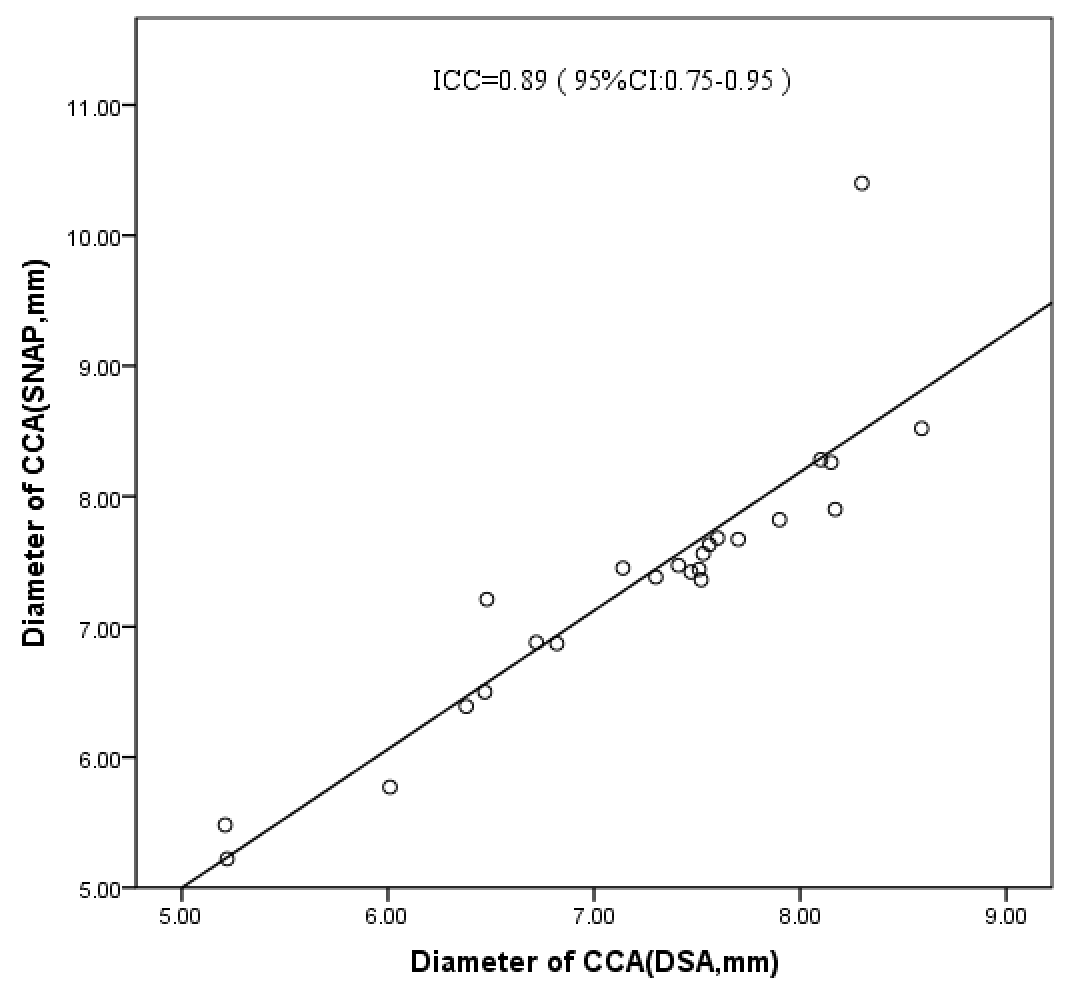

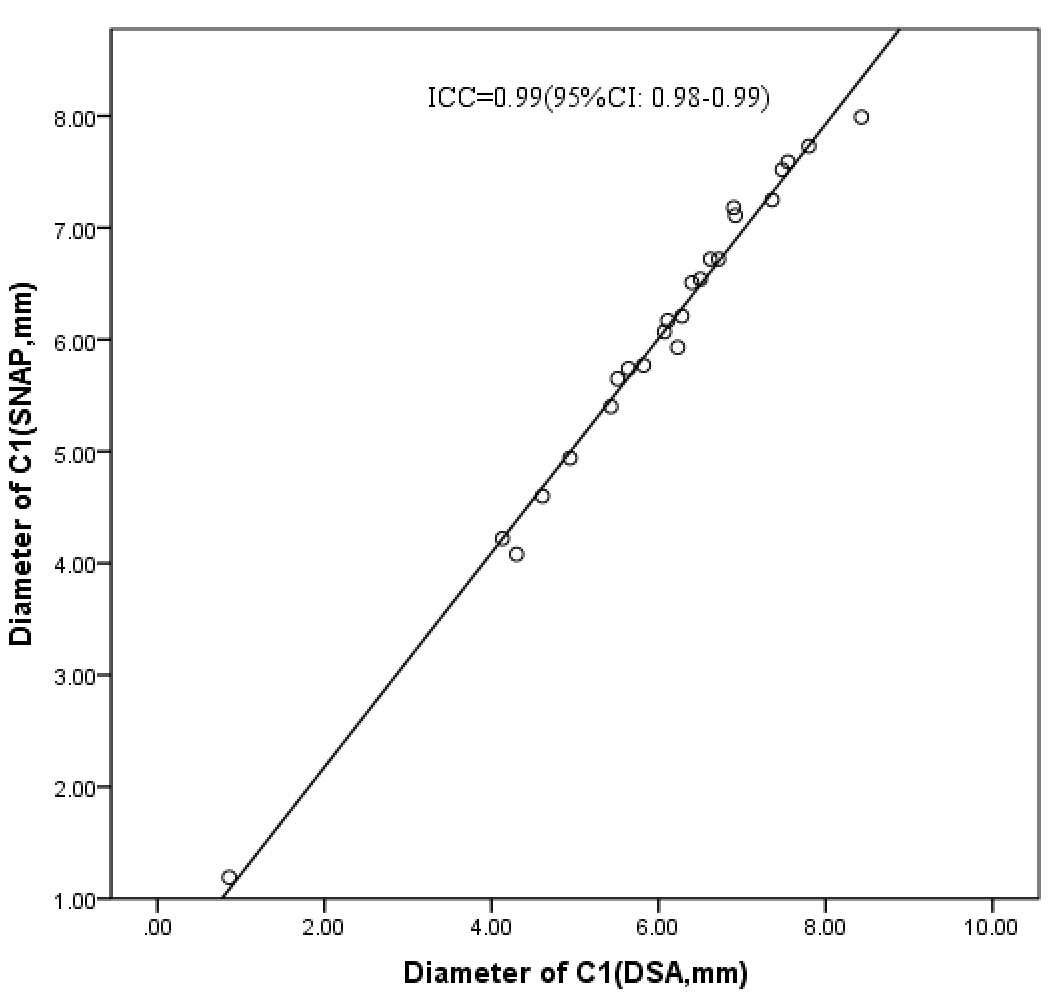

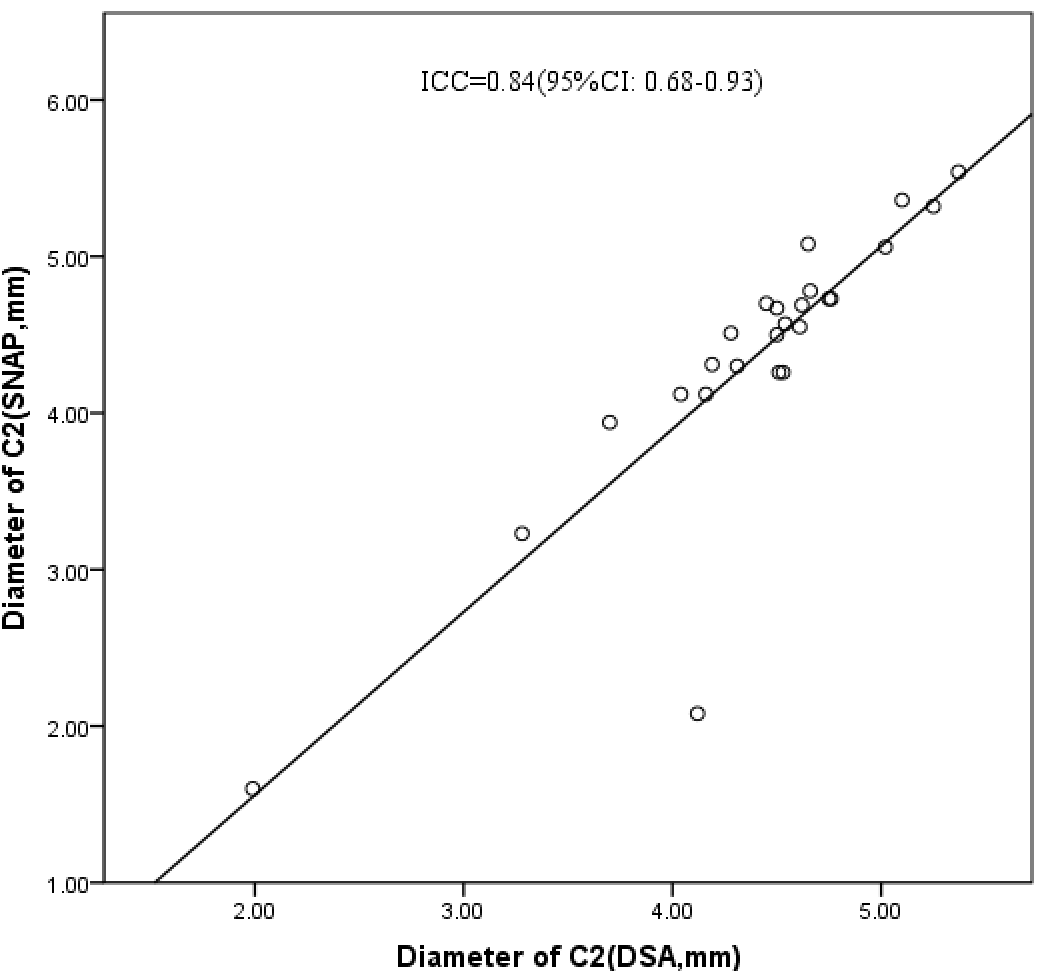

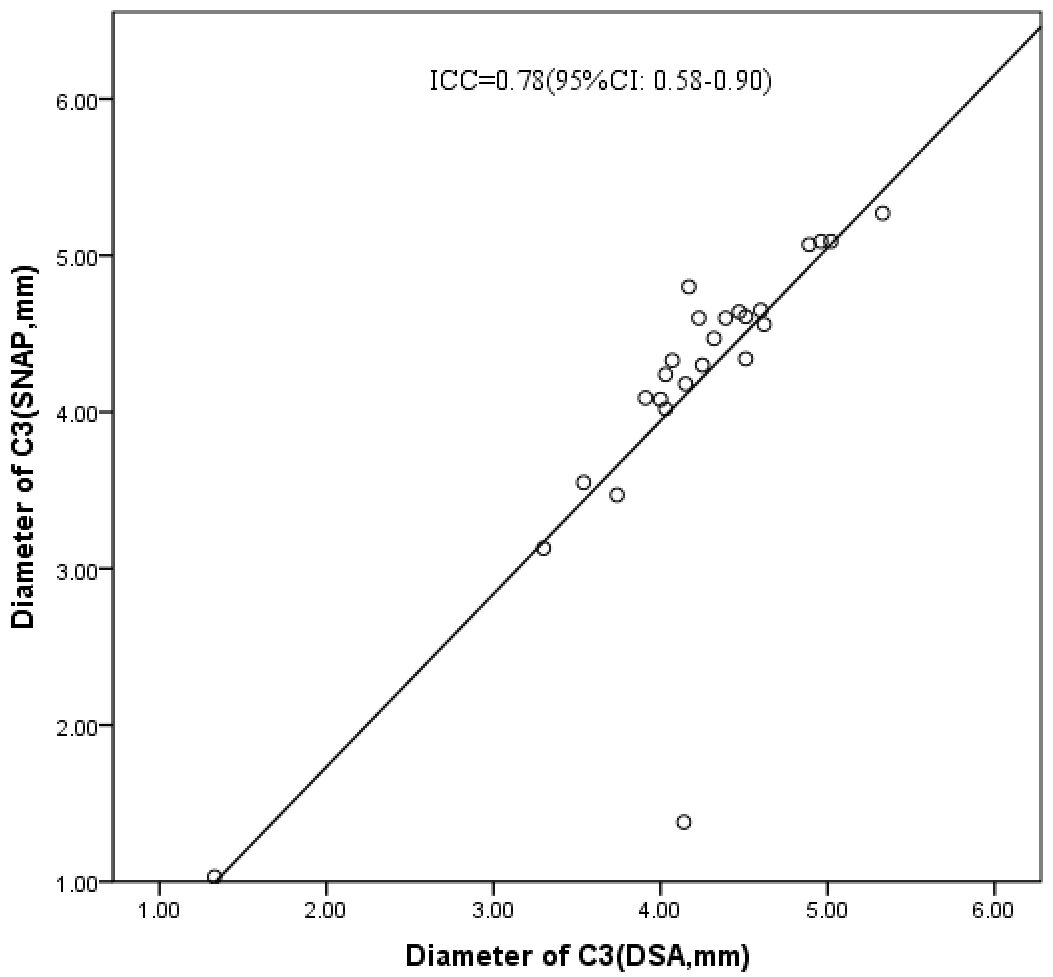

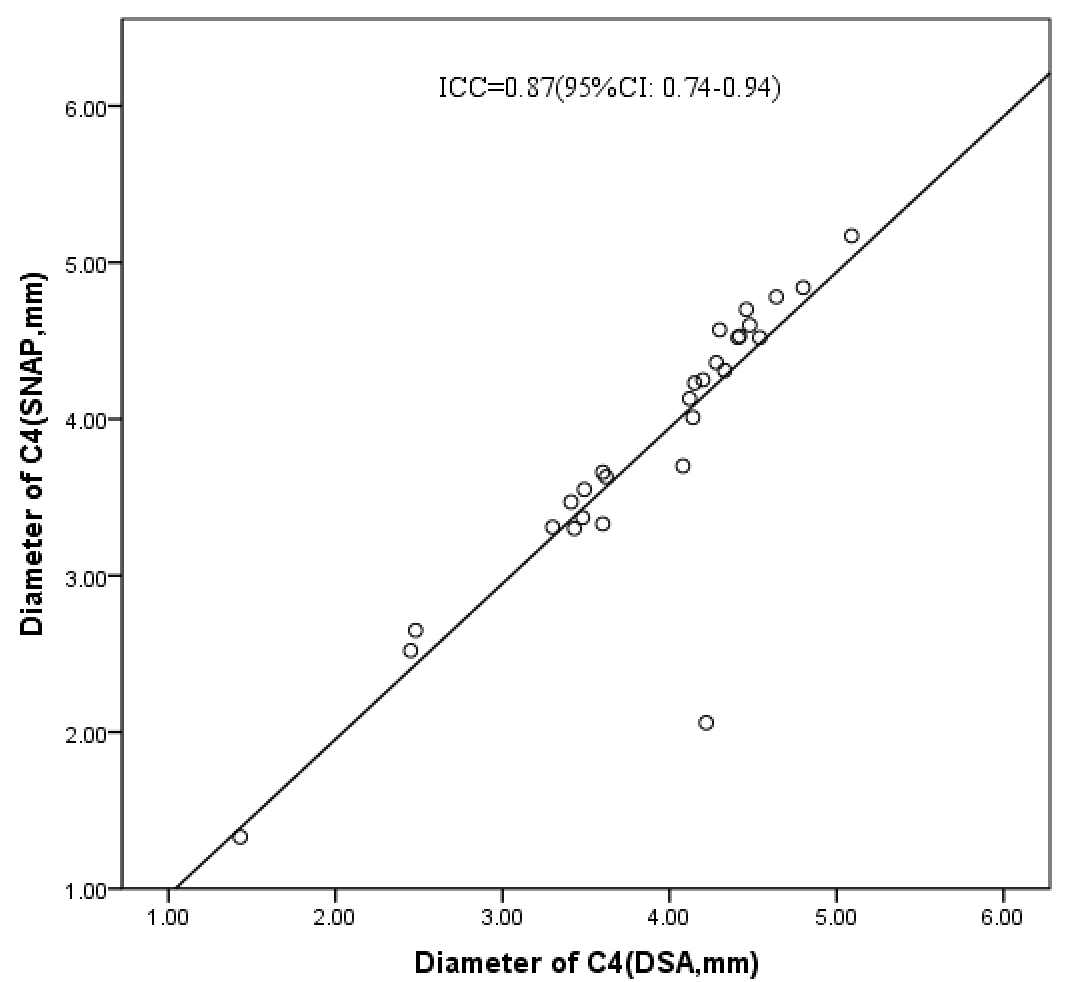

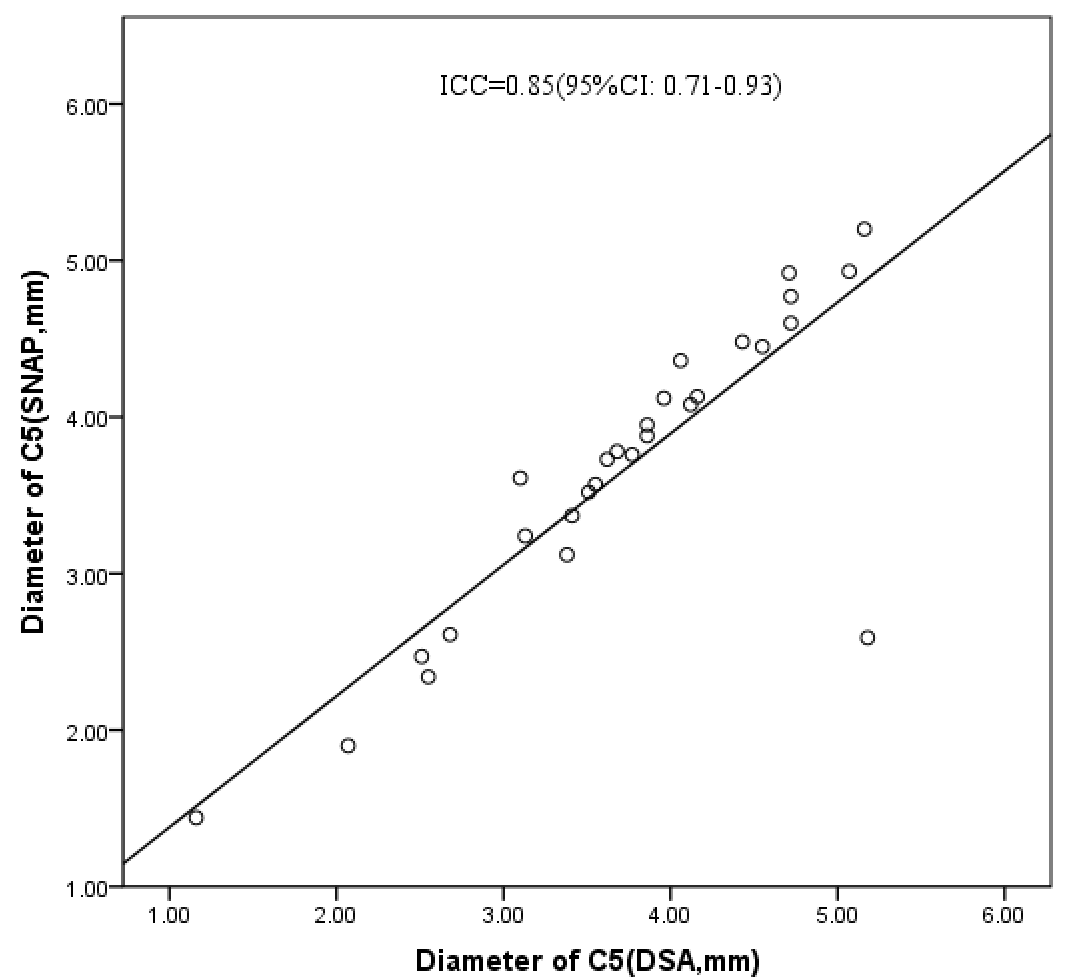

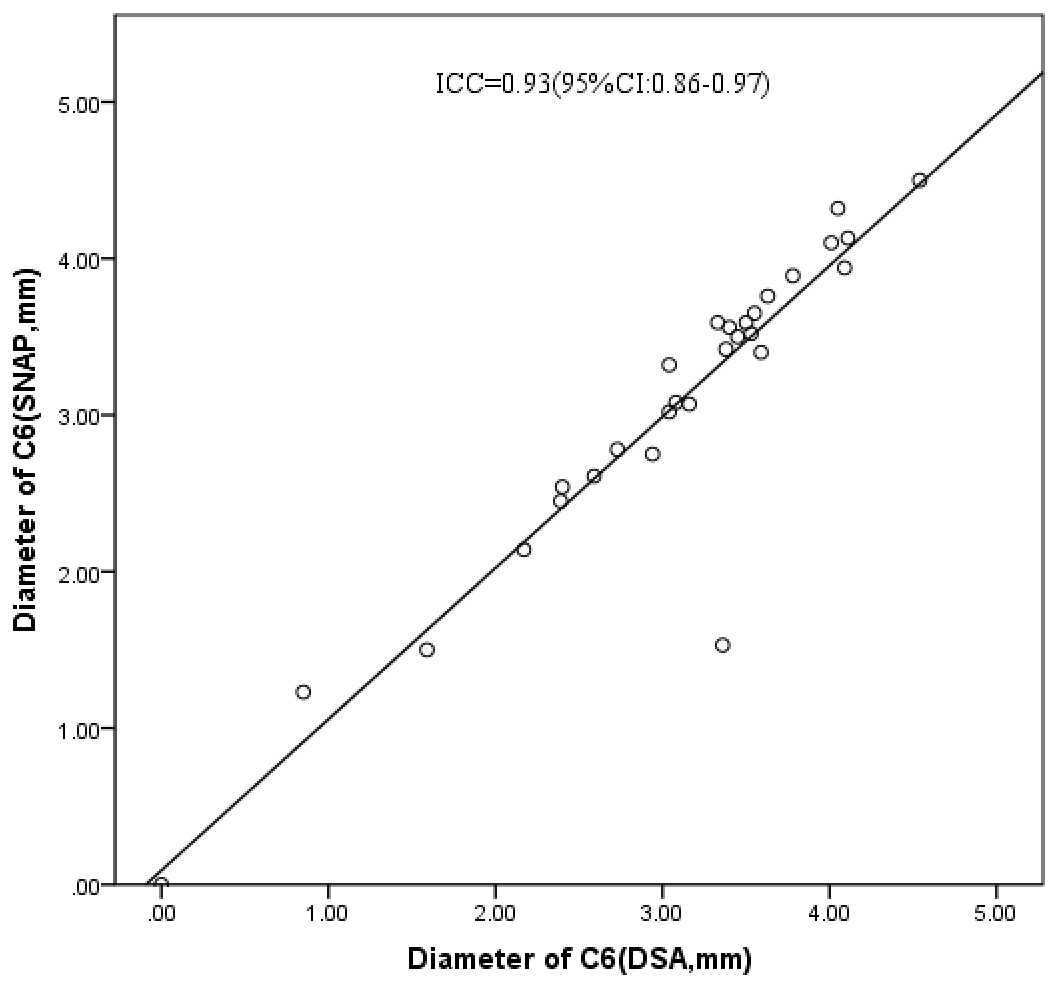

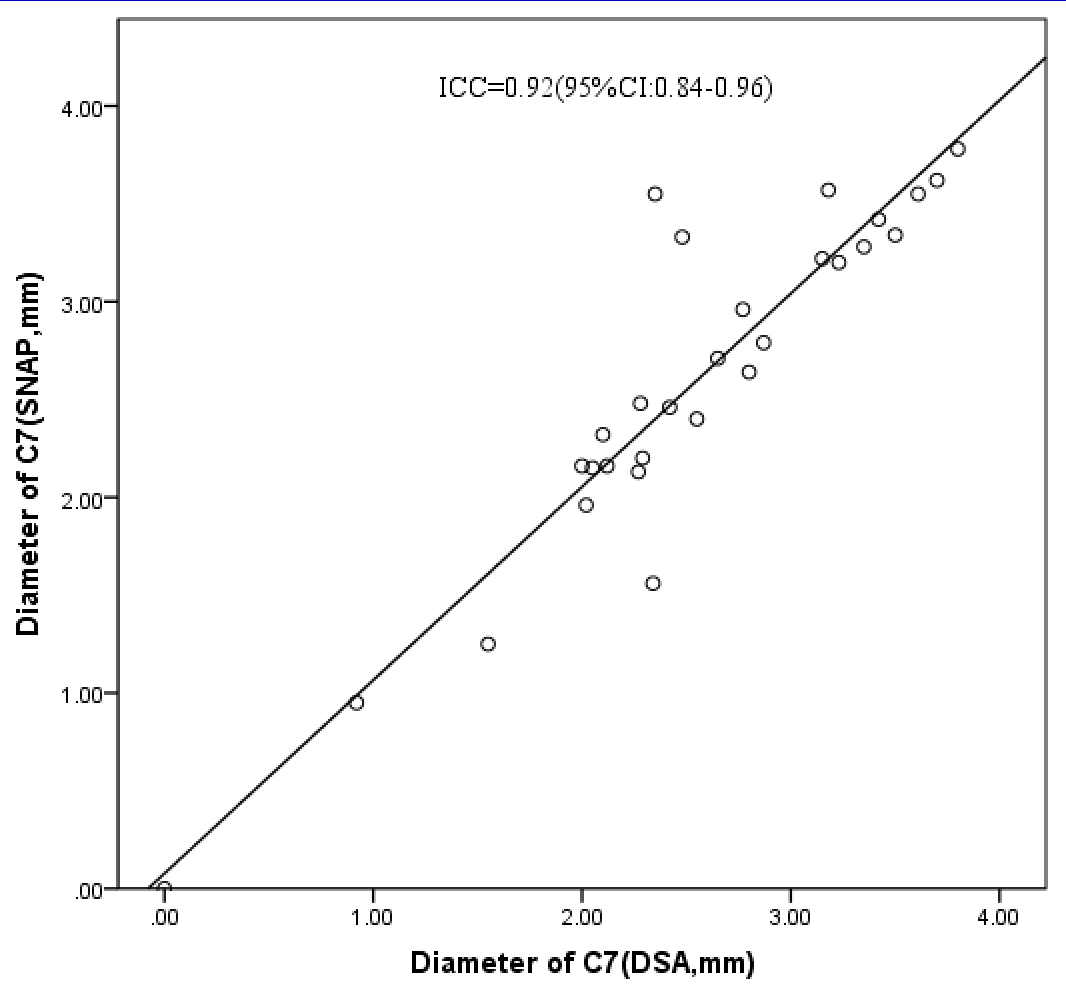

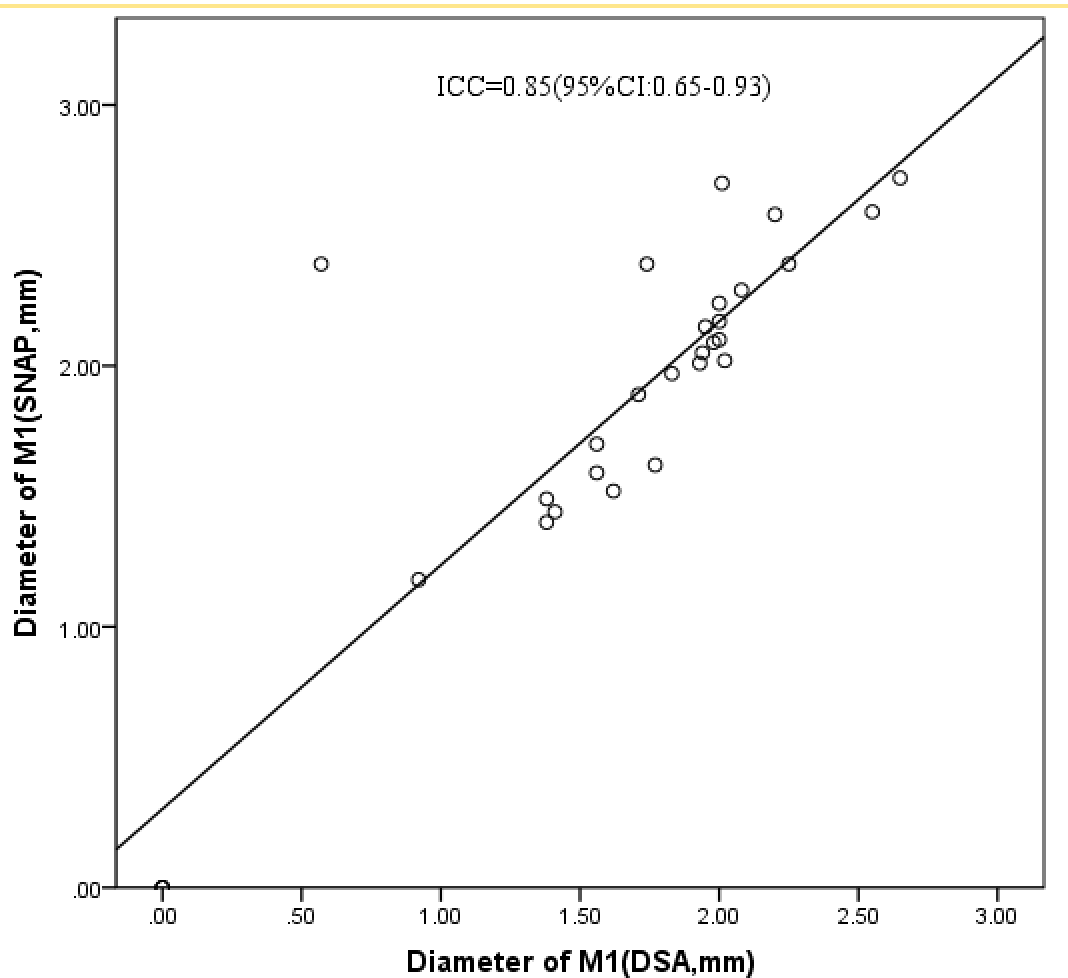

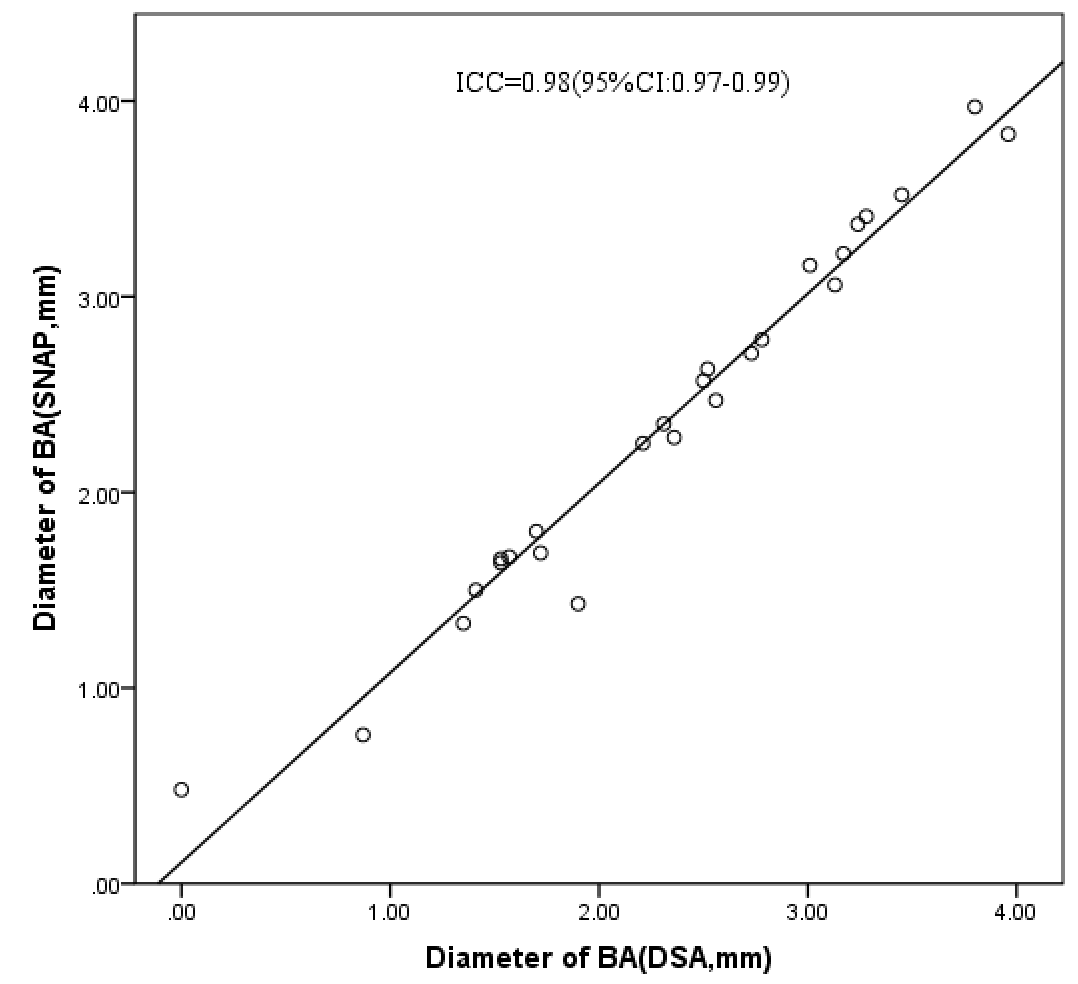

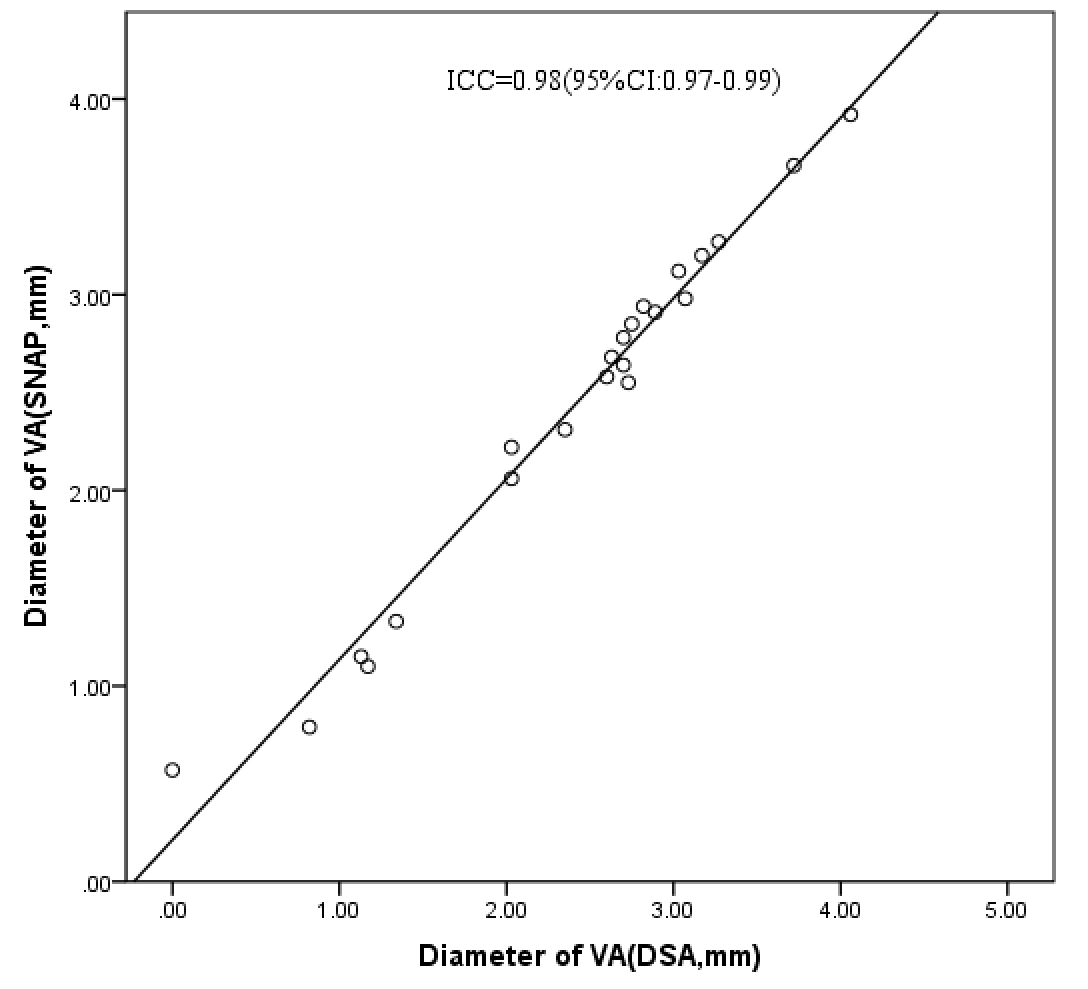


(B)


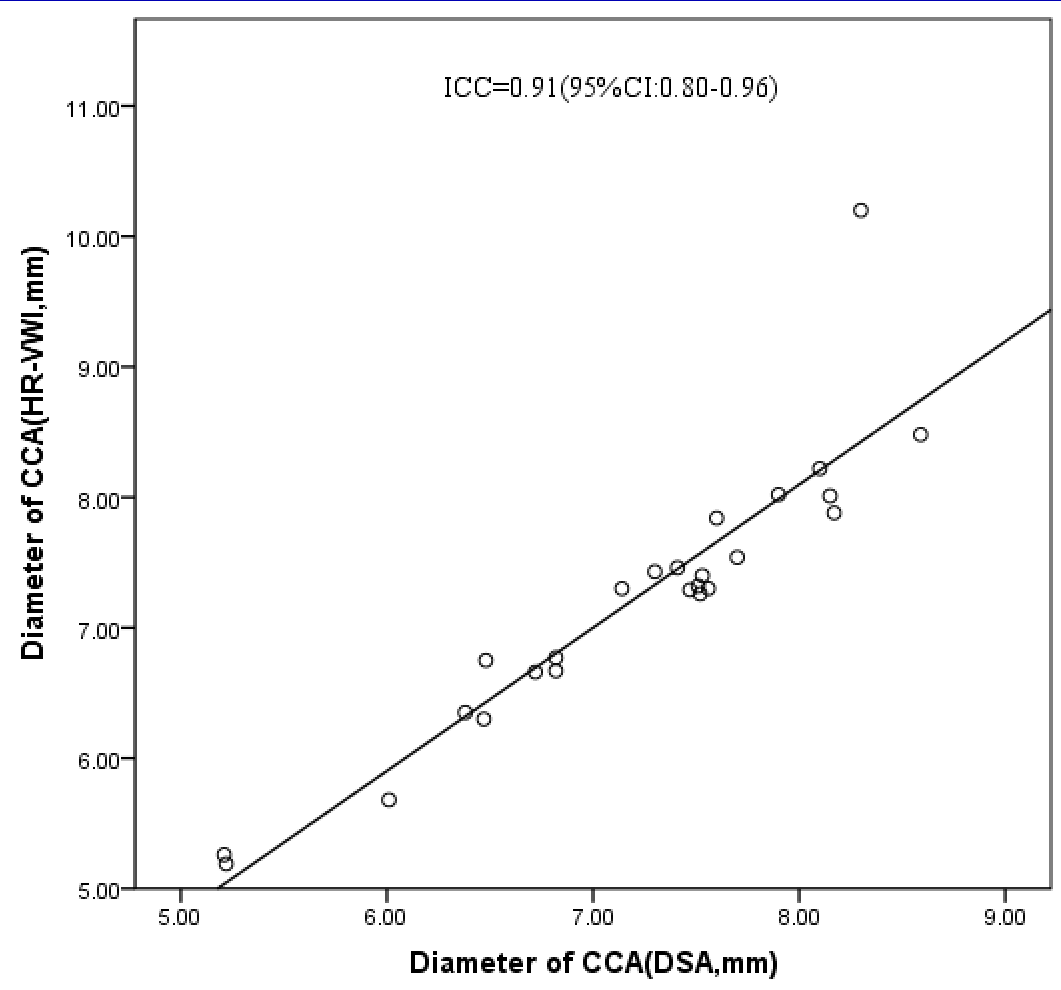

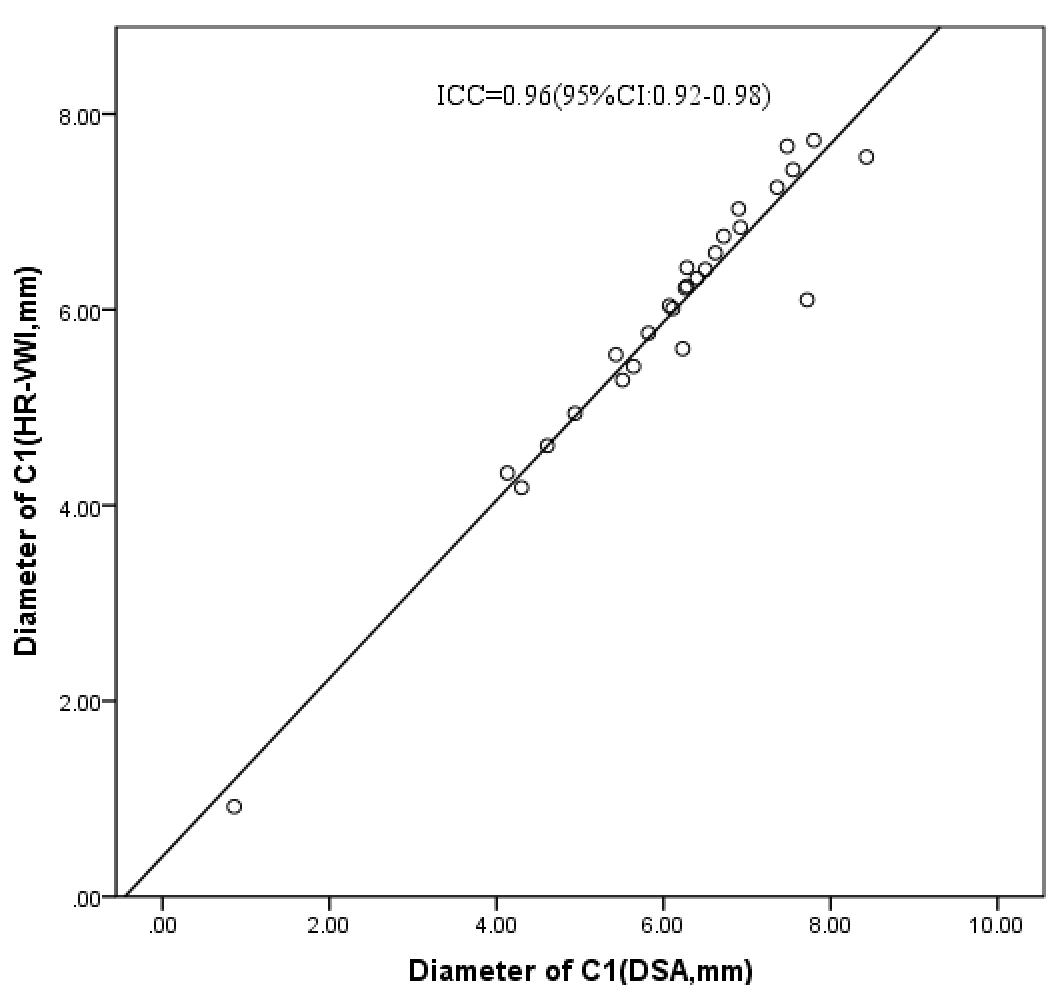

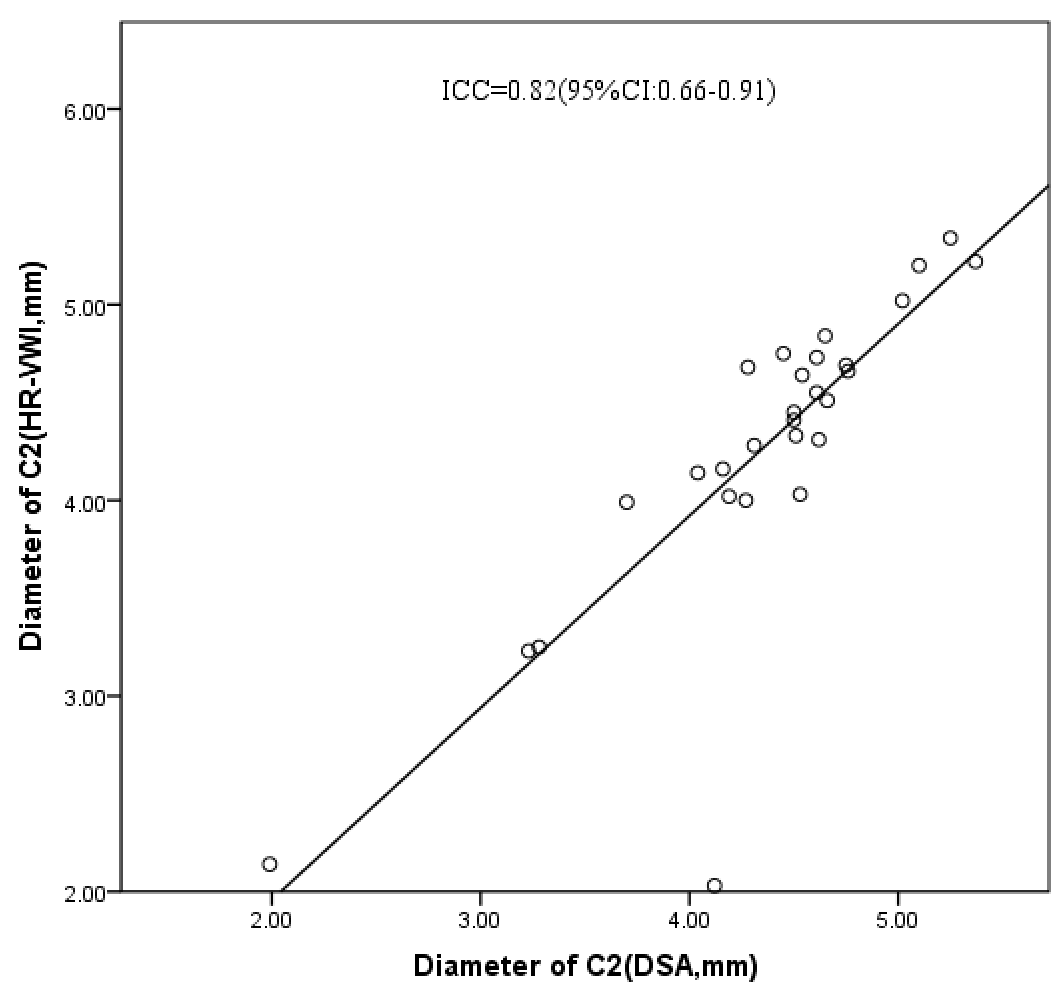

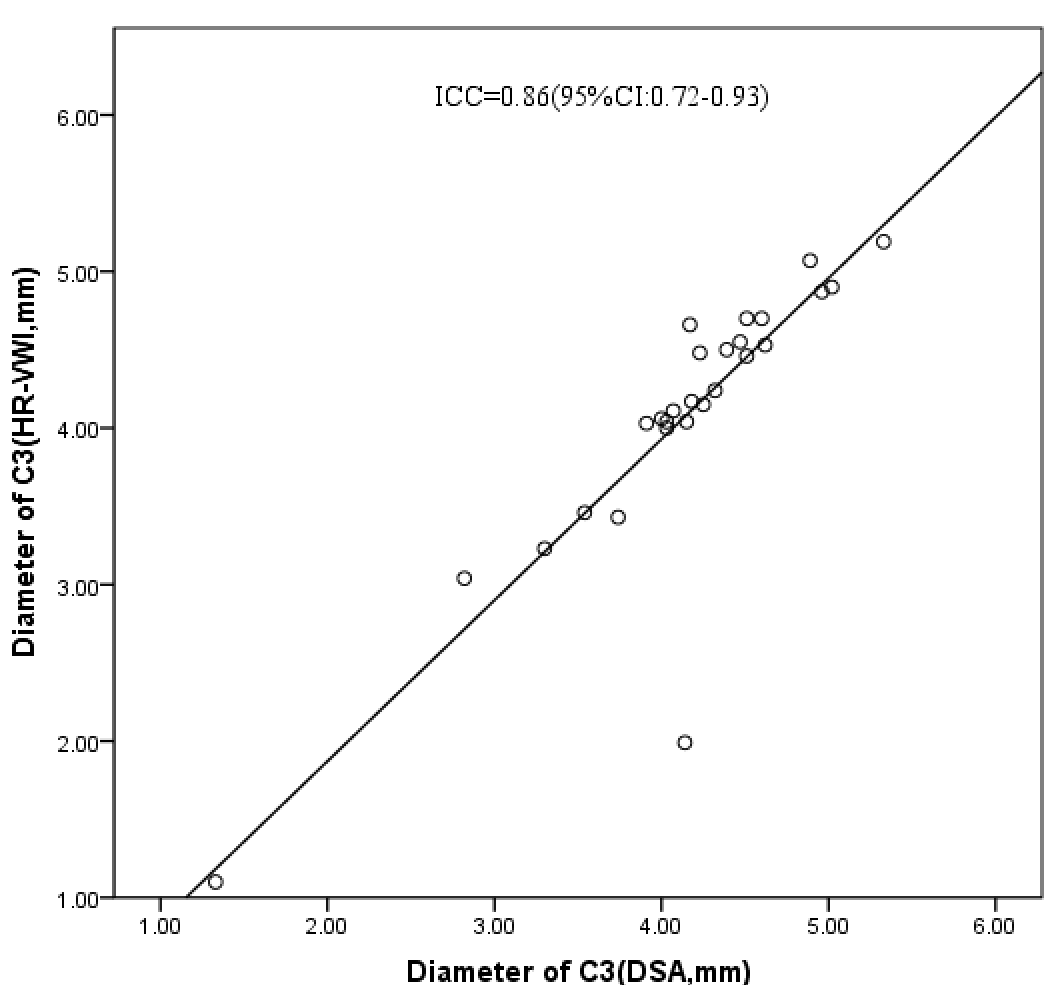

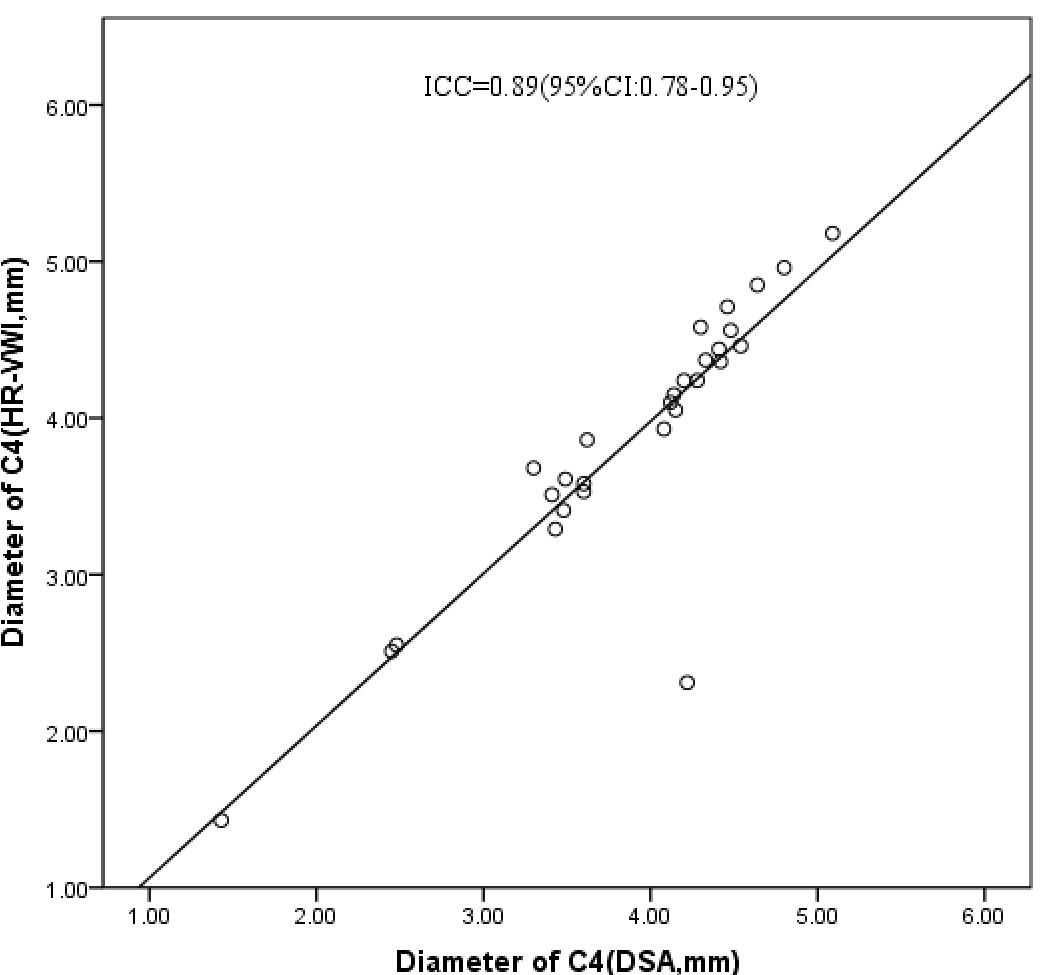

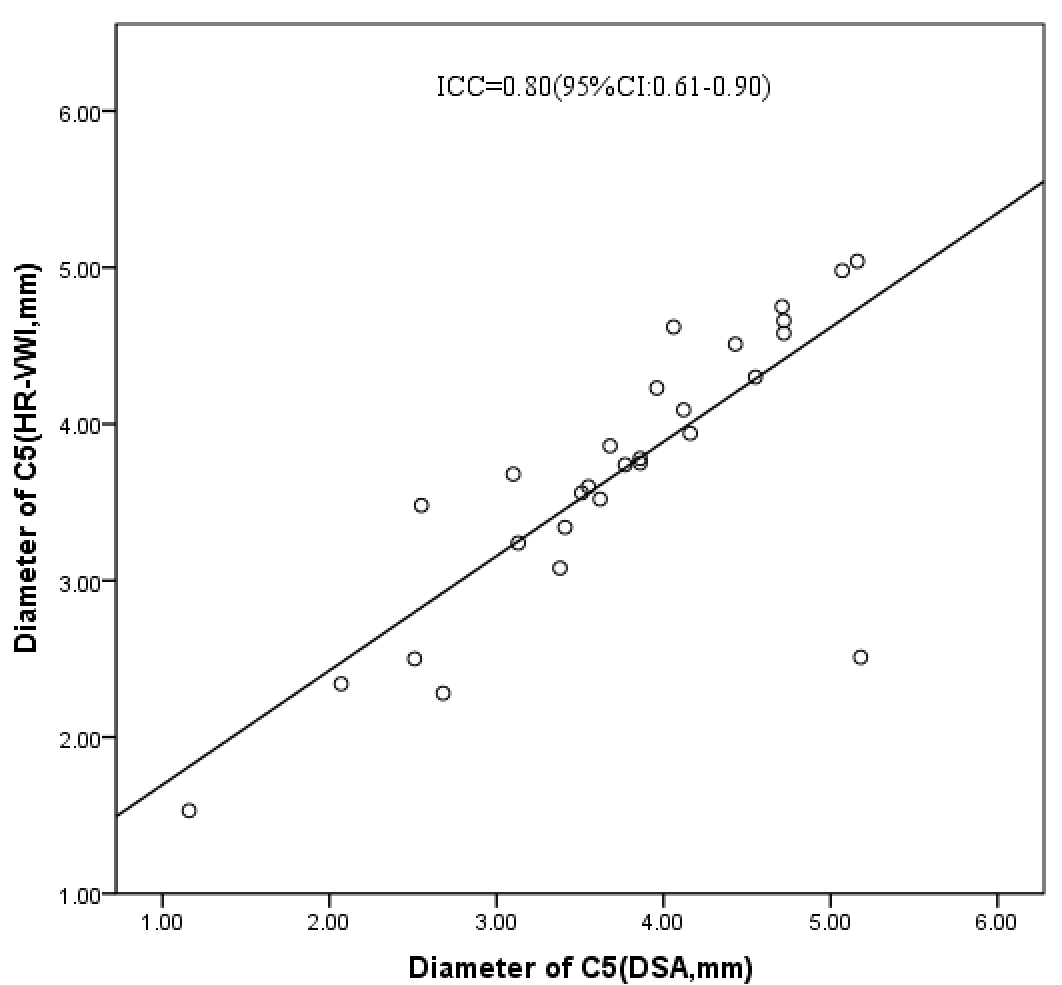

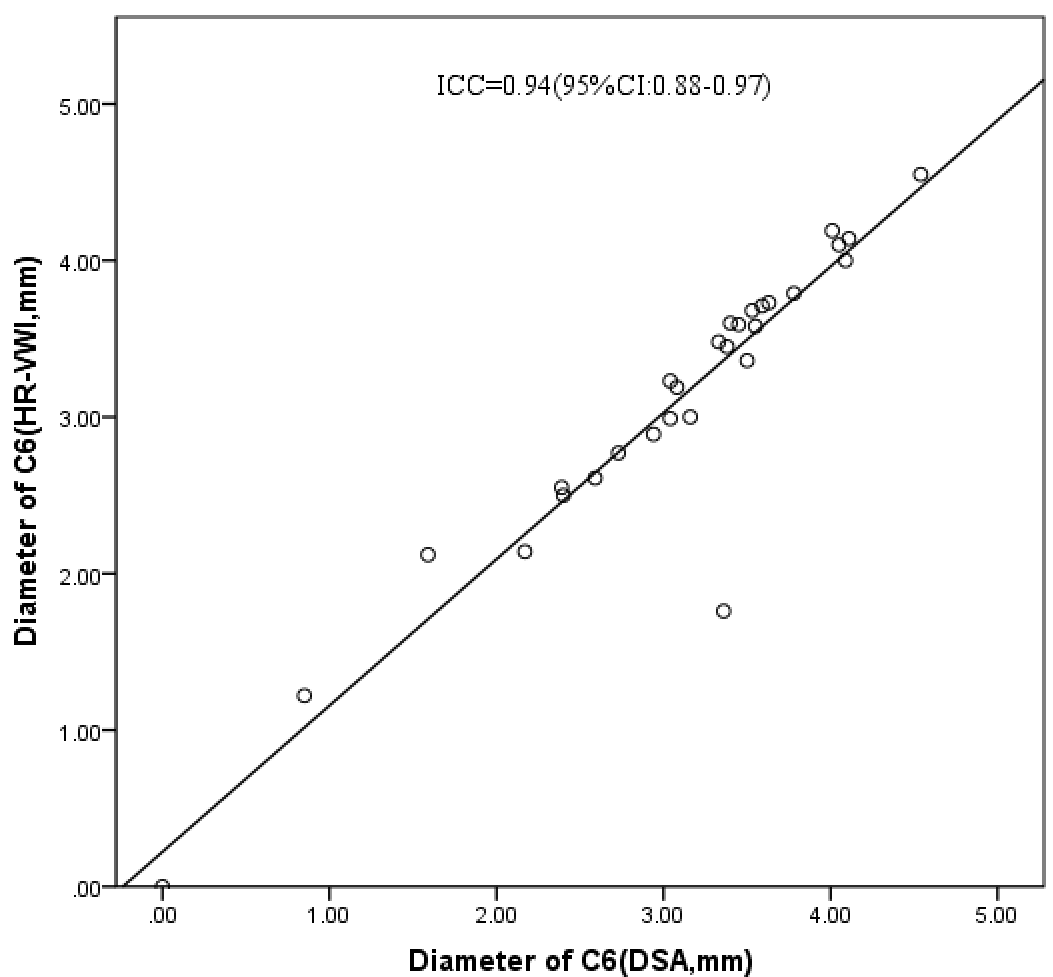

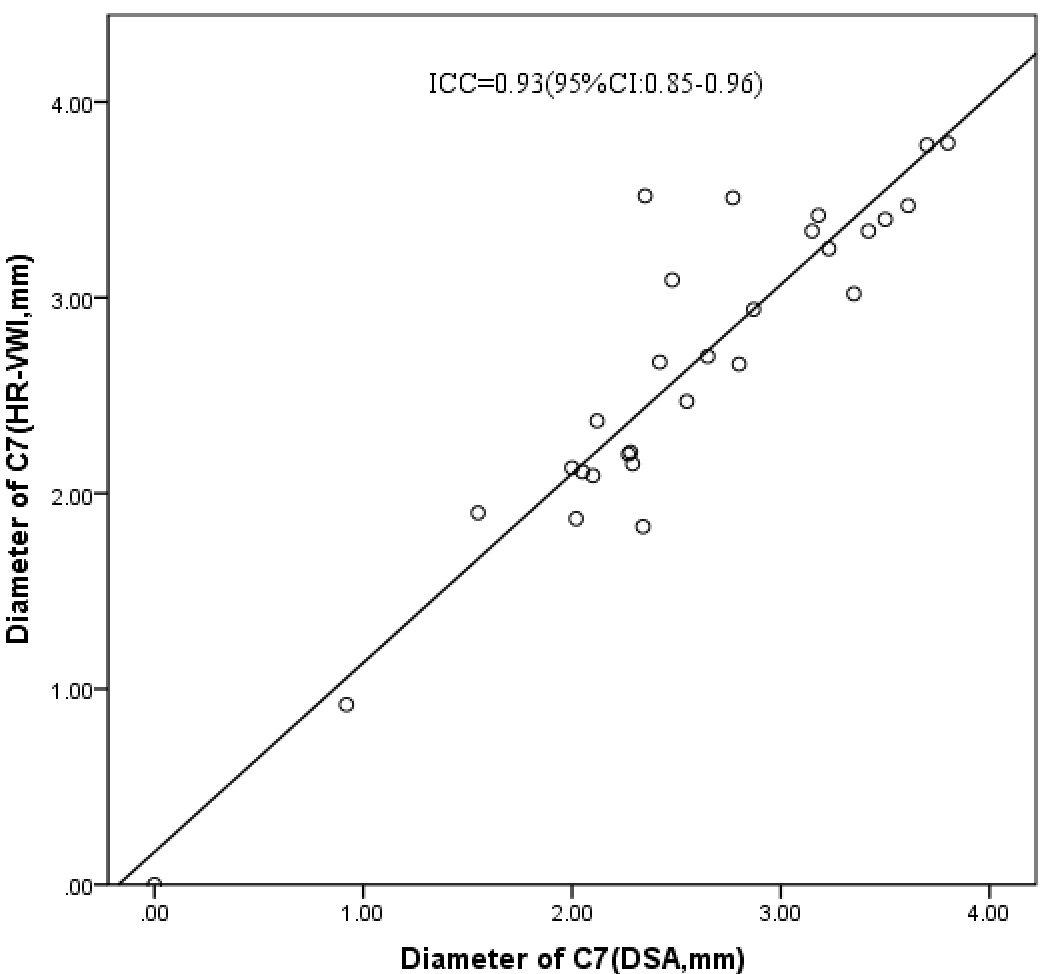

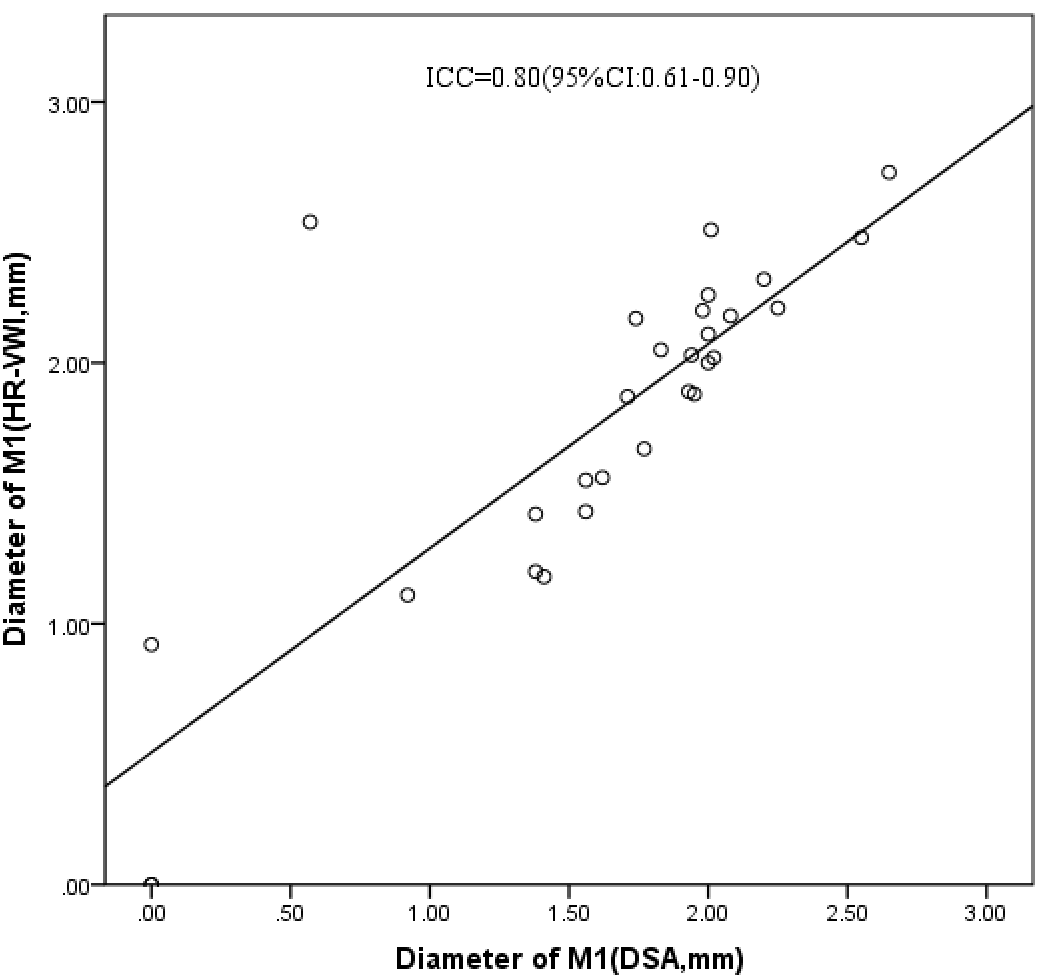

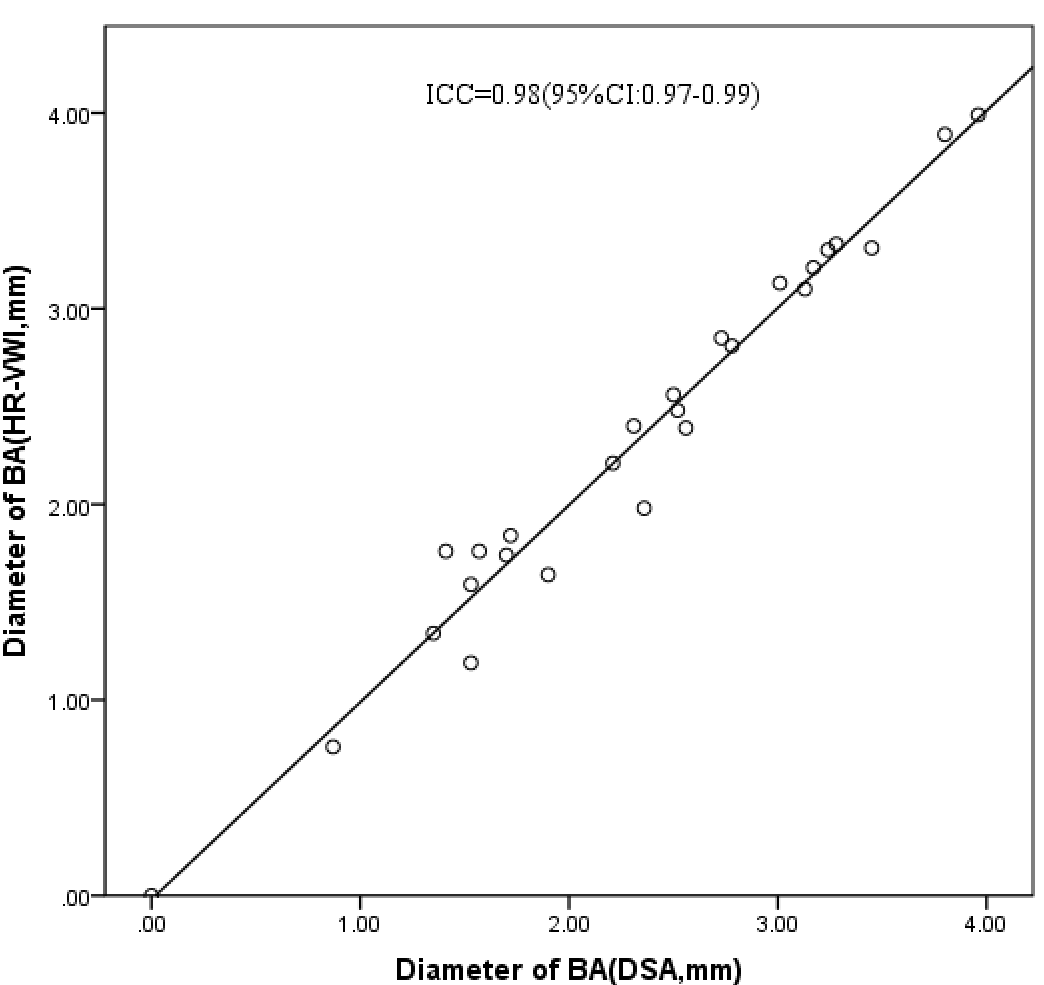

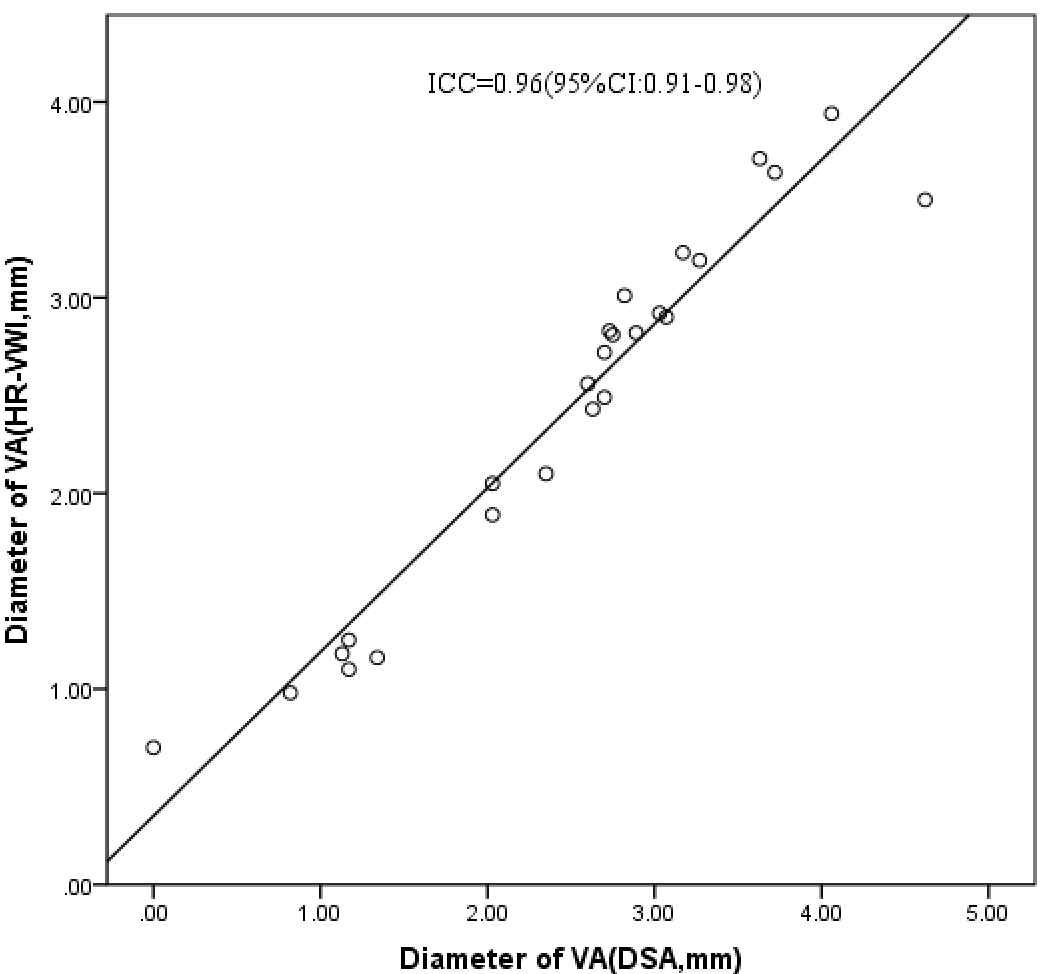

Supplement: Supplementary file 1 — Additional file 1. Table S1. The inter-group consistency of the simultaneous non-contrast angiography and intraplaque hemorrhage (SNAP) imaging in identifying intraplaque hemorrhage (IPH) at different sites of intracranial and carotid arteries. Fig. S1 The agreement between simultaneous non-contrast angiography and intraplaque hemorrhage (SNAP) and high-resolution vessel wall imaging (HR-VWI) with digital subtraction angiography (DSA) in measuring the diameter of different intracranial and carotid arterial segments. [file 12968_2022_849_MOESM1_ESM.docx]
